# Post-COVID symptom profiles and duration in a global convalescent COVID-19 observational cohort: Correlations with demographics, medical history, acute COVID-19 severity and global region

**DOI:** 10.7189/jogh.13.06020

**Published:** 2023-06-23

**Authors:** Shelly Karuna, Jorge A Gallardo-Cartagena, Deborah Theodore, Portia Hunidzarira, Juan Montenegro-Idrogo, Jiani Hu, Megan Jones, Vicky Kim, Robert De La Grecca, Meg Trahey, Carissa Karg, Azwi Takalani, Laura Polakowski, Julia Hutter, Maurine D Miner, Nathan Erdmann, Paul Goepfert, Rebone Maboa, Lawrence Corey, Katherine Gill, Shuying Sue Li, HVTN 405/HPTN 1901 Study Team

**Affiliations:** 1 Fred Hutchinson Cancer Center, Seattle, Washington, USA; 2 Centro de Investigaciones Tecnológicas, Biomédicas y Medioambientales, Universidad Nacional Mayor de San Marcos, Lima, Peru; 3 Columbia University Physicians & Surgeons, New York, New York, USA; 4 University of Zimbabwe Clinical Trials Research Centre, Harare, Zimbabwe; 5 Hutchinson Centre for Research in South Africa, Johannesburg, Republic of South Africa; ^6^National Institute of Allergy and Infectious Disease, Bethesda, Maryland, USA ^7^University of Alabama at Birmingham, Birmingham, Alabama, USA; 8 Ndlovu Research Centre, Elandsdoorn, Limpopo, Republic of South Africa; 9 Desmond Tutu HIV Foundation, University of Cape Town, Cape Town, Republic of South Africa

## Abstract

**Background:**

Post-COVID conditions are characterised by persistent symptoms that negatively impact quality of life after SARS-CoV-2 diagnosis. While post-COVID risk factors and symptoms have been extensively described in localised regions, especially in the global north, post-COVID conditions remain poorly understood globally. The global, observational cohort study HVTN 405/HPTN 1901 characterises the convalescent course of SARS-CoV-2 infection among adults in North and South America and Africa.

**Methods:**

We categorised the cohort by infection severity (asymptomatic, symptomatic, no oxygen requirement (NOR), non-invasive oxygen requirement (NIOR), invasive oxygen requirement (IOR)). We applied a regression model to assess correlations of demographics, co-morbidities, disease severity, and concomitant medications with COVID-19 symptom persistence and duration across global regions.

**Results:**

We enrolled 759 participants from Botswana, Malawi, South Africa, Zambia, Zimbabwe, Peru, and the USA a median of 51 (interquartile range (IQR) = 35-66) days post-diagnosis, from May 2020 to March 2021. 53.8% were female, 69.8% were 18-55 years old (median (md) = 44 years old, IQR = 33-58). Comorbidities included obesity (42.8%), hypertension (24%), diabetes (14%), human immunodeficiency virus (HIV) infection (11.6%) and lung disease (7.5%). 76.2% were symptomatic (NOR = 47.4%; NIOR = 22.9%; IOR = 5.8%). Median COVID-19 duration among symptomatic participants was 20 days (IQR = 11-35); 43.4% reported symptoms after COVID-19 resolution, 33.6% reported symptoms ≥30 days, 9.9% reported symptoms ≥60 days. Symptom duration correlated with disease severity (*P* < 0.001, NIOR vs NOR; *P* = 0.003, IOR vs NOR), lung disease (*P* = 0.001), race (*P* < 0.05, non-Hispanic Black vs White), and global region (*P* < 0.001). Prolonged viral shedding correlated with persistent abdominal pain (odds ratio (OR) = 5.51, *P* < 0.05) and persistent diarrhoea (OR = 6.64, *P* < 0.01).

**Conclusions:**

Post-COVID duration varied with infection severity, race, lung disease, and region. Better understanding post-COVID conditions, including regionally-diverse symptom profiles, may improve clinical assessment and management globally.

**Registration:**

Clinicaltrials.gov (#NCT04403880).

In early 2020, a pneumonia outbreak in Wuhan, Hubei Province, China, led to the identification of SARS-CoV-2, a novel betacoronavirus, as the causative agent of coronavirus disease 2019 (COVID-19) [1]. The term COVID-19 encompasses the broad clinical spectrum of symptomatic SARS-CoV-2 infection, ranging from asymptomatic infection to fulminant respiratory failure. Clinical features span general and respiratory [2,3], neurologic [4], gastrointestinal [5], and dermatologic [6] systems. Serious complications include acute respiratory failure, sepsis, thromboembolic complications [7,8] and death, which are often associated with age and comorbidities like hypertension, diabetes, and cardiovascular and chronic pulmonary disease [9,10].

Though not as variable as its clinical presentations, definitions of COVID-19 resolution have evolved globally throughout the pandemic. Most have focused on three primary features: absence of fever, a minimum timeframe from the onset of illness, and improving symptoms. The last criterion acknowledges that some symptoms persist after most have resolved and virus replication is undetectable.

Up to 80% of convalescent individuals may experience symptoms more than two weeks past acute infection, and some considerably longer [11-14]. The terminology and definitions of post-acute symptom persistence are not standardised and include long COVID, post-acute sequelae of SARS-CoV-2 (PASC), and post-COVID condition; definitions utilise symptom timeframes 1-6 months post-diagnosis, though have increasingly coalesced at around ≥3 months [15-17]. Persistent post-COVID symptoms include chest pain, fatigue, dyspnoea, and cognitive dysfunction [12-14,18]. Factors reportedly associated with persistent symptoms include older age, female sex assigned at birth, multiple comorbidities, and severe acute COVID-19 [19].

Several groups reported distinct acute COVID-19 profiles by global geographic location [20-24], yet most descriptions of post-COVID conditions originate from cohorts in North America, Europe, and East Asia [14,25,26]. The global post-COVID experience is poorly understood, with robust data from Africa and South America being particularly limited [12-14,27,28]. Small studies from Brazil reported symptoms at least three months post-diagnosis [29-31]. Similar reports from Africa, primarily in individuals with limited post-hospitalisation follow-up, described a significant post-COVID-19 burden and identified correlations with age, female sex, medical co-morbidities like hypertension, and COVID-19 severity [32-35].

During the first weeks of the pandemic, we initiated a prospective international cohort study at clinical research sites of the COVID-19 Prevention Network (CoVPN) in North and South America and sub-Saharan Africa funded by the National Institute of Allergy and Infectious Diseases (NIAID). We aimed to characterise the presentations of SARS-CoV-2 infection, including the clinical course of COVID-19, among convalescent individuals, measure adaptive immune responses to identify markers of COVID-19 disease severity and duration in a diverse population, and identify unique serologic reactivities that could differentiate SARS-CoV-2 infection from vaccination and provide a guidepost for characterising immune responses to candidate COVID-19 vaccines in early development at that time. Here we summarise our observations of the clinical course of COVID-19 over the first year post-diagnosis among study participants.

## METHODS

### Study cohort

From May 2020 through March 2021, 759 participants aged 18 years and older from the USA, Peru, Malawi, South Africa, Zambia, and Zimbabwe with a history of recent SARS-CoV-2 infection provided written informed consent and were enrolled into the HVTN 405/HPTN 1901 prospective observational cohort study at 53 clinical research sites (CRS) (Clinicaltrials.gov (#NCT04403880)).

We stratified participants by clinical presentation (symptomatic and asymptomatic disease, inpatient or outpatient care requirement) and age (18-55 or >55). Participants could be enrolled if they met the study eligibility criteria and the requirements of an age- and severity-based stratum that was enrolling at the time of their screening. Slots in each stratum were reserved for each global region to ensure balanced regional representation across age and disease severity characteristics. Enrolment preceded COVID-19 vaccine availability; no participants had received a COVID-19 vaccine at enrolment. See Tables S1 and S2 in the **Online Supplementary Document** for CRS listing, eligibility criteria, and schema.

Approval was granted by a central institutional review board (IRB) and, where applicable, by individual local IRBs (Table S3 in the **Online Supplementary Document**).

### Study procedures

All study procedures across the global clinical trial sites were harmonised through trial design (e.g. application of the same eligibility criteria, procedures, and monitoring), consistent training on study procedures (e.g. data collection methods), and regular communication across all sites throughout study implementation (e.g. bi-monthly calls to provide refresher training and share evolving understandings of COVID-19 epidemiology, diagnosis and management).

**Figure 1:**
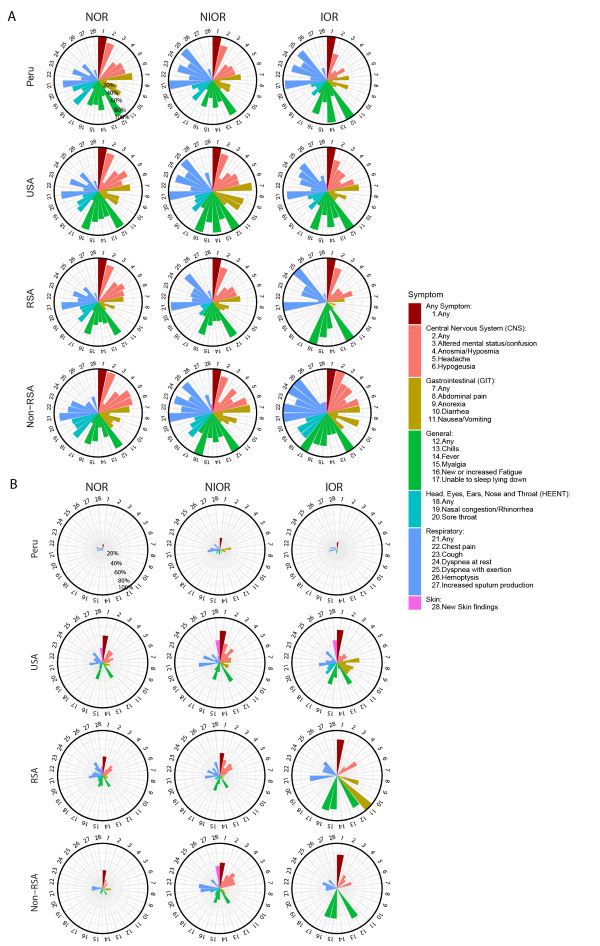
Clinical profiles by region and symptom severity. **Panel A.** Proportion of participants with each symptom, by region and symptom severity. **Panel B.** Rates of persistence of COVID-19 symptoms after acute COVID-19 resolution, by region and symptom severity. Each plot shows proportion or rate of response, from 0% (middle of pie chart) to 100% (outer ring of pie chart). NOR – no oxygen requirement, NIOR – non-invasive oxygen requirement, IOR – infection requiring invasive oxygen, RSA – Republic of South Africa.

Participants provided nasopharyngeal and blood samples and demographic and medical information at enrolment and two, four, and 12 months post-enrolment. We solicited twenty-one symptoms across five systems at baseline and follow-up (**Figure 1**). For each symptom, CRSs reported onset, total duration (days) and whether, in their judgement and informed by local guidance, the symptom(s) persisted after COVID-19 resolution (Y/N). Details of each participant’s clinical course were also reported, including hospitalisation status, oxygen requirement, and receipt of concomitant medications.

### Statistical methods

We summarised participants’ characteristics at enrolment and by region, and compared across regions using a χ^2^ test for categorical variables and the Kruskal-Wallis test for continuous variables. Factors of interest included COVID-19 severity, prolonged viral shedding, clinical co-morbidities, body mass index (BMI, ≥30 vs <30), region and demographics, including age (>55 vs 18-55), sex assigned at birth, and race/ethnicity (non-Hispanic Black, Hispanic/Latino, Others vs non-Hispanic White). We constructed a separate regression model to assess the correlation of each factor of interest with symptom duration (days) and symptom persistence after acute COVID-19 resolution (Y/N), adjusting for confounders (COVID-19 severity, age, sex assigned at birth and region). We tested overall correlations of COVID-19 severity and region using the Wald test. When assessing the correlation of a factor included among confounders, that factor was not adjusted.

We used Firth logistic regressions [36] for correlations with symptom persistence (Y/N), and loglinear regressions for correlations with symptom duration (days) and for correlations of concomitant medications with symptom duration. We calculated adjusted *P*-values (q-values) to control for false positive findings involving multiple comparisons using the Benjamini and Hochberg method [37]. We reported odds ratios (ORs), 95% confidence intervals (CIs), *P*-values, and q-values for correlations with specific symptoms and with symptom persistence, and geometric mean ratios (GMRs), 95% CIs, *P*-values, and q-values for correlations with symptom duration. We considered *P*-values ≤0.05 and q-values ≤0.2 as statistically significant.

We generated Kaplan-Meier (K-M) curves of symptom duration by COVID-19 severity and by region and compared mean symptom duration using the two-sample comparison method of restricted mean survival time [38] implemented in the *survRM2* R package.

## RESULTS

### Demographics and correlations with post-COVID symptom profiles

We enrolled 759 participants from Peru (n = 191, 25.2%), USA (n = 197, 26.0%), Republic of South Africa (RSA) (n = 286, 37.7%), and non-RSA Sub-Saharan Africa (n = 85, 11.2%): 53.8% were female, of whom 69.8% were 18-55 years old (median = 44, interquartile range (IQR) = 33-58). Participants identified as non-Hispanic Black (42.7%), Hispanic (27.9%), non-Hispanic White (15.8%), or Other Race (13.6%). Age, sex assigned at birth, and race/ethnicity differed significantly by region (**Table 1**).

**Table 1:** Participant characteristics at baseline overall and by region*

	Total (n = 759)	Peru (n = 191)	USA (n = 197)	RSA (n = 286)	Non-RSA (n = 85)	*P*-value
**Age**						
Mean (SD)	45.1 (14.83)	48.4 (14.73)	48 (15.64)	42 (14.14)	41.2 (12.36)	<0.001
Median (IQR)	44 (33-58)	47 (36-60.5)	51 (34-60)	40 (31-54)	40 (32-49)	
Range	18-86	18-81	18-86	18-84	19-71	
18-55	530 (69.8)	118 (61.8)	115 (58.4)	228 (79.7)	69 (81.2)	<0.001
55+	229 (30.2)	73 (38.2)	82 (41.6)	58 (20.3)	16 (18.8)	
**Sex assigned at birth**						
Female	408 (53.8)	82 (42.9)	94 (47.7)	185 (64.7)	47 (55.3)	<0.001
Male	351 (46.2)	109 (57.1)	103 (52.3)	101 (35.3)	38 (44.7)	
**BMI**						
Mean (SD)	30 (7.43)	28.1 (4.55)	30.1 (7.29)	31.7 (8.86)	28.2 (6.28)	<0.001
Median (IQR)	28.7 (24.9-33.8)	27.3 (25-30.6)	29 (24.5-33.7)	30.5 (25.4-37)	28 (23.9-31.6)	
Range	14.3-65.1	15.6-45.2	19.3-55	14.3-65.1	16.6-47.3	
<30	429 (56.5)	133 (69.6)	107 (54.3)	132 (46.2)	57 (67.1)	<0.001
>30	325 (42.8)	58 (30.4)	90 (45.7)	149 (52.1)	28 (32.9)	
**Days since SARS-CoV-2 diagnosis**						
Mean (SD)	51.5 (20.64)	45.4 (17.84)	58.6 (19.63)	49.2 (21.91)	56.7 (18.76)	<0.001
Median (IQR)	50.5 (35-66)	43 (32-57)	60 (46-71)	49 (32-63.5)	57 (42-69)	
Range	5-185	13-127	13-131	5-185	15-105	
<28	86 (11.3)	28 (14.7)	9 (4.6)	46 (16.1)	3 (3.5)	<0.001
28-42	171 (22.5)	56 (29.3)	32 (16.2)	66 (23.1)	17 (20.0)	
42-56	167 (22)	53 (27.7)	39 (19.8)	58 (20.3)	17 (20.0)	
>56	332 (43.7)	54 (28.3)	117 (59.4)	113 (39.5)	48 (56.5)	
**COVID-19 severity**						
Asymptomatic	181 (23.8)	41 (21.5)	45 (22.8)	77 (26.9)	18 (21.2)	0.001
Symptomatic, no O_2_ (NOR)	360 (47.4)	81 (42.4)	93 (47.2)	144 (50.3)	42 (49.4)	
Symptomatic, O_2_, no intubation (NIOR)	174 (22.9)	45 (23.6)	48 (24.4)	60 (21.0)	21 (24.7)	
Symptomatic, intubation (IOR)	44 (5.8)	24 (12.6)	11 (5.6)	5 (1.7)	4 (4.7)	
**Prolonged viral shedding**						
No	718 (94.6)	180 (94.2)	172 (87.3)	285 (99.7)	81 (95.3)	<0.001
Yes	41 (5.4)	11 (5.8)	25 (12.7)	1 (0.3)	4 (4.7)	
**Current cigarettes/marijuana**						
No	683 (90.0)	184 (96.3)	164 (83.2)	252 (88.1)	83 (97.6)	<0.001
**Smoker**						
Yes	76 (10.0)	7 (3.7)	33 (16.8)	34 (11.9)	2 (2.4)	
**Ever cigarettes/marijuana**						
No	516 (68.0)	136 (71.2)	84 (42.6)	220 (76.9)	76 (89.4)	<0.001
**Smoker**						
Yes	243 (32.0)	55 (28.8)	113 (57.4)	66 (23.1)	9 (10.6)	
**Hypertension**						
No	577 (76.0)	163 (85.3)	132 (67.0)	215 (75.2)	67 (78.8)	<0.001
Yes	182 (24.0)	28 (14.7)	65 (33.0)	71 (24.8)	18 (21.2)	
**COPD/emphysema/asthma**						
No	702 (92.5)	182 (95.3)	166 (84.3)	271 (94.8)	83 (97.6)	<0.001
Yes	57 (7.5)	9 (4.7)	31 (15.7)	15 (5.2)	2 (2.4)	
**Diabetes**						
No	653 (86.0)	170 (89.0)	166 (84.3)	240 (83.9)	77 (90.6)	0.217
Yes	106 (14.0)	21 (11.0)	31 (15.7)	46 (16.1)	8 (9.4)	
**HIV**						
No	671 (88.4)	176 (92.1)	168 (85.3)	249 (87.1)	78 (91.8)	0.115
Yes	88 (11.6)	15 (7.9)	29 (14.7)	37 (12.9)	7 (8.2)	
**Race/ethnicity**						
Hispanic-Latino/a	212 (27.9)	191 (100.0)	20 (10.2)	1 (0.3)	-	<0.001
Black-Non-Hispanic	324 (42.7)	-	51 (25.9)	205 (71.7)	68 (80.0)	
White-Non-Hispanic	120 (15.8)	-	111 (56.3)	9 (3.1)	-	
Other	103 (13.6)	-	15 (7.6)	71 (24.8)	17 (20.0)	

SD – standard deviation, IQR – interquartile range, RSA – Republic of South Africa, BMI – body mass index, NOR – no oxygen requirement, NIOR – non-invasive oxygen requirement, IOR – invasive oxygen requirement, COPD – chronic obstructive pulmonary disease, HIV – human immunodeficiency virus

*Values are n (%) unless otherwise specified.

Few demographic factors correlated with symptom persistence (i.e. persistence beyond acute COVID-19 resolution; Y/N) or with symptom duration (i.e. absolute number of days of any symptom). Overall, non-Hispanic Blacks were about half as likely as Whites to report persistence of symptoms (**Table 2**). With respect to specific symptom classes, persistence of general symptoms (e.g. myalgia, fatigue) correlated with non-Hispanic Black vs non-Hispanic White race; non-Hispanic Blacks were about half as likely to report persistent general symptoms and even less likely to report persistent respiratory symptoms (Table S5 in the **Online Supplementary Document**). In the Americas, only, symptom duration correlated with older age (Table S4 in the **Online Supplementary Document**).

**Table 2:** Associations of COVID-19 severity, medical co-morbidities, and demographics with persistence beyond acute COVID-19 resolution and duration of any symptoms among all symptomatic participants

	Persistence beyond acute COVID-19 resolution					Symptom duration			
Comparison*	**OR**	**95% CI**	***P*-value**	**q-value†**	**GMR**		**95% CI**	***P*-value**	**q-value†**
COVID-19 Severity (overall test)	-	-	0.017‡	0.057‡	-		-	<0.001‡	<0.001‡
NIOR vs NOR	1.67‡	1.13-2.48‡	0.011‡	0.041‡	1.48‡		1.27-1.72‡	<0.001‡	<0.001‡
IOR vs NOR	1.96	0.94-4.07	0.071	0.141	1.5‡		1.14-1.96‡	0.003‡	0.01‡
IOR vs NIOR	1.17	0.55-2.5	0.679	0.764	1.01		0.77-1.34	0.923	0.923
Age (>55 vs 18-55)	1.14	0.76-1.7	0.525	0.745	1.12		0.96-1.31	0.136	0.261
Sex at birth (male vs female)	0.92	0.63-1.33	0.647	0.764	0.92		0.8-1.06	0.24	0.368
BMI (≥30 vs <30)	1.08	0.74-1.56	0.698	0.764	1.1		0.95-1.27	0.19	0.312
COPD/emphysema/asthma	1.83	0.96-3.58	0.067	0.141	1.55‡		1.21-1.98‡	0.001‡	0.002‡
Diabetes	1.16	0.71-1.92	0.55	0.745	1.05		0.86-1.28	0.636	0.731
Hypertension	1.11	0.72-1.7	0.641	0.764	0.93		0.79-1.11	0.43	0.55
HIV/AIDS	0.84	0.49-1.44	0.526	0.745	1.03		0.83-1.27	0.818	0.855
Prolonged viral shedding	1.98	0.94-4.28	0.074	0.141	1.17		0.88-1.55	0.279	0.401
Current cigarettes/marijuana smoker	0.79	0.41-1.51	0.475	0.745	0.89		0.69-1.15	0.374	0.506
Ever cigarettes/marijuana smoker	1.33	0.87-2.02	0.191	0.338	1.02		0.87-1.2	0.779	0.853
Non-Hispanic Black vs Non-Hispanic White	0.46‡	0.23-0.9‡	0.024‡	0.069‡	0.81		0.62-1.06	0.129	0.261
Others vs Non-Hispanic White	1.09	0.54-2.21	0.819	0.819	1.11		0.84-1.47	0.456	0.552
Region (overall test)	-	-	<0.001‡	<0.001‡	-		-	<0.001‡	<0.001‡
USA vs Peru	8.95‡	5.25-15.72‡	<0.001‡	<0.001‡	1.85‡		1.53-2.24‡	<0.001‡	<0.001‡
Non-RSA vs RSA	1.07	0.61-1.86	0.814	0.819	1.32‡		1.05-1.66‡	0.02‡	0.05‡
RSA vs Peru	4.35‡	2.59-7.51‡	<0.001‡	<0.001‡	1.14		0.95-1.38	0.15	0.266
Non-RSA vs Peru	4.65‡	2.44- 9.02‡	<0.001‡	<0.001‡	1.51‡		1.18-1.92‡	0.001‡	0.004‡
RSA vs USA	0.49‡	0.31-0.75‡	0.001‡	0.005‡	0.62‡		0.52-0.74‡	<0.001‡	<0.001‡
Non-RSA vs USA	0.52‡	0.29-0.94‡	0.029‡	0.074‡	0.81		0.64-1.04	0.093	0.214

NOR – no oxygen requirement; NIOR – non-invasive oxygen requirement, IOR – invasive oxygen requirement, RSA – Republic of South Africa, CI – confidence interval, OR – odds ratio, GMR – geometric mean ratio, BMI – body mass index, HIV – human immunodeficiency virus, AIDS – acquired immunodeficiency syndrome, COPD – chronic obstructive pulmonary disease

*Adjusted for a set of confounders (COVID-19 severity, age, sex assigned at birth, and region). For COVID-19 severity, age, sex assigned at birth, and region, the comparison was adjusted for the rest of confounders. Overall test for COVID-19 severity and region was done using Wald-test.

†Adjusted for multiple comparisons to control false discovery rate.

‡Values for significant with *P*-value ≤0.05 and q-value ≤0.2.

### Baseline clinical co-morbidities and correlations with post-COVID symptom profiles

Comorbidities at baseline included obesity (42.8%), hypertension (24%), diabetes (14%), human immunodeficiency virus (HIV) infection (11.6%), lung disease (i.e. chronic obstructive pulmonary disease, emphysema, and/or asthma; 7.5%), ever smoking (32%), and currently smoking (10%). Overall, only lung disease significantly correlated with symptom duration; participants with lung disease reported 55% longer symptom duration than those without lung disease (**Table 2**). However, the prevalence of most clinical comorbidities differed significantly by location (**Table 1**); in regional analyses, the correlation of symptom duration with lung disease remained statistically significant only in the Americas (Table S4 in the **Online Supplementary Document**).

We also evaluated correlations of co-morbidities with persistence and duration of individual symptom systems. Overall, participants reporting persistent general symptoms (e.g. fatigue, myalgia) were nearly three times as likely to have a history of lung disease and nearly twice as likely to have a history of smoking (Table S5 in the **Online Supplementary Document**). Furthermore, participants with a history of lung disease reported 45%-58% longer duration of general, neurologic and respiratory symptoms.

In the Americas, symptom duration also correlated with a BMI≥30 (Table S4 in the **Online Supplementary Document**). Over all participants, BMI≥30 correlated with a longer duration of respiratory symptoms (Table S4 in the **Online Supplementary Document**).

### Concomitant medications and correlations with post-COVID symptom profiles

Overall, 456 of 759 (60.1%) participants took medications during acute COVID-19; the proportion who took medications, number of medications taken, and the most common medications varied by region (Table S6 in the **Online Supplementary Document**).

Use of any concomitant medication correlated with about 30% longer duration of any symptom (Table S7 in the **Online Supplementary Document**). Some medication classes correlated with distinct symptom duration profiles; for example, use of corticosteroids alone did not correlate with symptom duration, but use of other immunomodulators correlated with about 30% longer duration of any symptom and of respiratory symptoms, specifically (Table S7 in the **Online Supplementary Document**).

### Symptom profiles, COVID-19 severity and rates of post-COVID clinical presentations

Overall, general (92%), respiratory (88%), neurologic (86%), gastrointestinal (69%) and head, eyes, ears, nose, and throat (HEENT, 60%) symptoms were most common across all regions; dermatologic (7%) symptoms were less common (**Figure 1**, panel A; Tables S8 and S9 in the **Online Supplementary Document**).

The range of SARS-CoV-2 infection severity included asymptomatic infection (n = 181, 23.8%), symptomatic infection with no oxygen requirement (NOR) (n = 360, 47.4%), symptomatic infection with non-invasive oxygen requirement (NIOR) (n = 174, 22.9%), and symptomatic infection requiring invasive oxygen (IOR) (n = 44, 5.8%), e.g. intubation. Severity differed by region (*P* = 0.001); for example, NOR rates were highest in RSA (50.3%) and lowest in Peru (42.4%), while IOR rates were highest in Peru (12.6%) and lowest in RSA (1.7%) (**Table 1**).

Among the 578 symptomatic participants, the median duration of symptoms was 20 days (IQR = 11-35); 43.4% had ≥1 persistent symptom after COVID-19 resolution; 33.6% reported ≥1 symptom at ≥30 days; and 9.9% reported ≥1 symptom at ≥60 days.

### Correlations of post-COVID symptom profiles with COVID-19 severity

**Figure 2:**
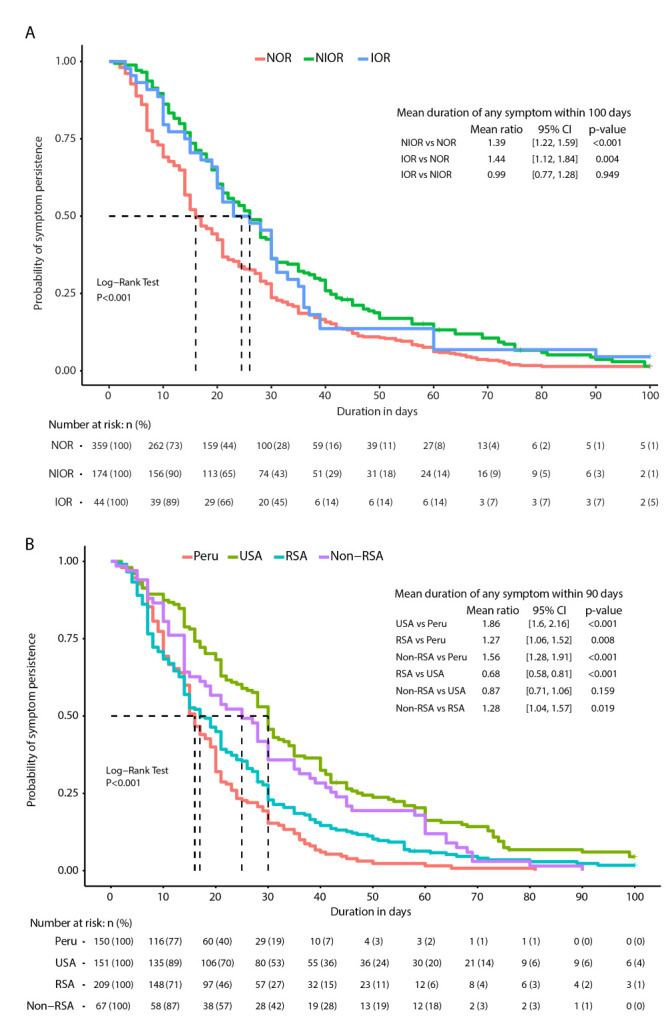
Kaplan-Meier (K-M) curves of symptom duration and the comparison of restricted mean duration. **Panel A.** Between severity groups. **Panel B.** Between regions. NOR – no oxygen requirement, NIOR – non-invasive oxygen requirement, IOR – infection requiring invasive oxygen, RSA – Republic of South Africa.

Among symptomatic participants, an oxygen requirement (i.e. greater severity) correlated with 40% longer mean symptom duration within 100 days post-enrolment vs. NOR, longer median symptom duration (16 days NOR vs 26 days NIOR + IOR, *P* < 0.001), higher rates of symptom persistence (40% NOR vs 49.1% NIOR + IOR, *P* < 0.05), higher rates of symptoms ≥30 days (27.9% NOR vs 43.1% NIOR + IOR, *P* < 0.001), and higher rates of symptoms ≥60 days (7.5% NOR vs 13.8% NIOR + IOR, *P* < 0.05) (**Figure 2**, panel A; Figures S1 and S2, and Table S9 in the **Online Supplementary Document**). We found no significant difference in duration by invasive vs non-invasive oxygen requirement (**Figure 2****,** panel A and Figure S2 in the **Online Supplementary Document**), but participants who required oxygen during COVID-19 experienced 50% greater symptom duration than those with no oxygen requirement (**Table 2**).

These observations were consistent across symptom classes regarding both persistence beyond acute COVID-19 resolution and total symptom duration. General symptoms were more persistent among individuals who required oxygen than among those who did not; 89.4% of individuals with NOR, 93.7% of individuals with NIOR, and 100% of individuals with IOR reported general symptoms (*P* < 0.05) for a median NOR of 13 (IQR = 7-22), NIOR of 15 (IQR = 10-30), or IOR of 19 days (IQR = 9.5-31) (*P* < 0.001) (Table S9 in the **Online Supplementary Document**). Greater severity correlated with over three times the odds of persistent general symptoms (IOR vs NOR: OR = 3.38, 95% CI = 1.45-8.01; *P* < 0.01, q <0.02) and participants requiring oxygen reported 43%-70% longer general symptom duration. Individuals requiring oxygen also had increased odds of persistent respiratory symptoms and about 60% longer respiratory symptom duration, and had increased odds of neurologic symptoms with 40%-53% longer neurologic symptom duration than those not requiring oxygen, after adjusting for age, sex assigned at birth, and region (Table S5 in the **Online Supplementary Document**). Neurologic symptoms were less common among those who required invasive oxygen (NOR = 88.6%, NIOR = 83.9%, IOR = 70.5%; *P* < 0.01). However, in participants with neurologic symptoms, the duration was significantly longer among those with an oxygen requirement compared to those without (median 10 days NOR vs 14 days NIOR vs 13 days IOR, *P* < 0.01) (Table S9 in the **Online Supplementary Document**).

In regional analyses, the correlation of symptom duration with severity was stronger in the Americas than in Africa (Table S4 in the **Online Supplementary Document**). Though rates of symptom persistence were low in Peru irrespective of symptom severity, rates were higher in those who required oxygen (NIOR and IOR) than in those who did not (NOR) in Peru, as in all regions evaluated (**Figure 1**, panel B).

### Correlations of post-COVID symptom profiles with region

Symptom persistence and duration strongly correlated with region (**Figure 1**, panel B and **Figure 2**, panel B; Figure S1 in the **Online Supplementary Document**). Among participants who reported symptom persistence beyond acute COVID-19 (n = 251, 43.44%), the rates were lowest in Peru (n = 26, 17.3%), followed by RSA (n = 95, 45.5%), non-RSA Africa (n = 32, 47.8%) and USA (n = 98, 64.5%) (Table S8 in the **Online Supplementary Document**). Participants in the USA were nearly nine times more likely than those in Peru to report symptoms persisting beyond acute COVID-19 resolution and reported a total symptom duration nearly twice that in Peru (**Table 2**).

African participants were over four times more likely than Peruvian participants to report symptom persistence, but about half as likely as USA participants (**Table 2**). No significant difference in the likelihood of symptom persistence beyond acute COVID-19 resolution was observed in a comparison of participants within Africa, though the overall absolute duration of any symptom was about 30% longer in African countries outside RSA compared to RSA (**Table 2**).

Median symptom duration was also lower in Peru than in all other regions (Figure S1 and Table S8 in the **Online Supplementary Document**). The median symptom duration was 20 days (IQR = 11-35) overall, 16 (IQR = 10-24) in Peru, 17 (IQR = 8-30) in RSA, 25 (IQR = 14-43) in non-RSA African countries, and 30 (IQR = 16-47) in the USA (*P* < 0.001). Among all participants, 163 (28%) reported any symptom persisting for ≥30 days and 46 (8%) reported any symptom persisting for ≥60 days. These rates varied by region (*P* < 0.001) (Table S8 in the **Online Supplementary Document**), ranging from 15.3% in Peru to 45.4% in USA reporting symptom persistence for >30 days and 1.3% in Peru to 15.8% in USA reporting symptom persistence for >60 days.

An analysis by restricted mean symptom duration adjusting for age, sex assigned at birth and severity reflected a similar pattern: compared to Peru, the mean symptom duration was 1.86 times longer in the USA, 1.56 times longer in African countries outside of South Africa, and 1.27 times longer in the Republic of South Africa (**Figure 2**, panel B).

These trends were observed across multiple body systems (**Figure 1****,** panel B and Figure S1 in the **Online Supplementary Document**). For example, persistence of general symptoms was much more common in USA vs Peru (OR = 20.77; 95% CI = 8.81-59.63, *P* < 0.001, q <0.001), in RSA vs Peru (OR = 13.84; 95% CI = 5.81-40.01, *P* < 0.001, q <0.001), and in non-RSA vs Peru (OR = 10.91; 95% CI = 4.07-33.87, *P* < 0.001, q < 0.001) (Table S5 in the **Online Supplementary Document**). Furthermore, general symptom duration (e.g. fever, myalgia, fatigue) was longer in USA vs Peru, and non-RSA vs Peru and shorter in RSA vs USA and non-RSA vs USA (Table S5 in the **Online Supplementary Document**). Comparable trends were observed with neurologic, gastrointestinal and respiratory symptoms.

### Prolonged viral shedding

**Figure 3:**
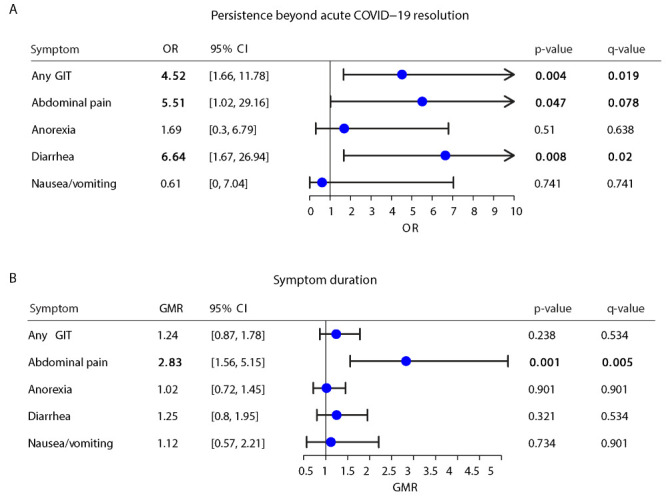
Correlation of prolonged viral shedding with duration and persistence of gastrointestinal symptoms. GIT – gastrointestinal tract, OR – odds ratio, GMR – geometric mean ratio.

In all, 5.4% of all participants had prolonged viral shedding, defined as a report of two tests detecting virus at least 21 days apart; most people with prolonged viral shedding were enrolled in the USA (**Table 1**). Prolonged viral shedding correlated with persistence of any gastrointestinal symptoms (OR = 4.52; 95% CI = 1.66-11.78, *P* < 0.01, q <0.02) (**Figure 3**) beyond acute COVID-19 resolution. Specifically, prolonged viral shedding correlated with nearly three times longer duration of abdominal pain (GMR = 2.83; 95% CI = 1.56-5.15, *P* = 0.001, q = 0.01) and with over five times the odds of persistent abdominal pain (OR = 5.51; 95% CI = 1.02-29.16, *P* < 0.05, q <0.1) and over six times the odds of persistent diarrhoea (OR = 6.64; 95% CI = 1.67-26.94, *P* < 0.01, q <0.05) (**Figure 3**).

## DISCUSSION

Our longitudinal cohort of over 750 individuals in Peru, the USA, and sub-Saharan and Eastern Africa shows patterns of COVID-19 symptom duration and persistence that varied with infection severity, race, a history of lung disease, and global region. Correlations of post-COVID clinical syndromes with demographic and clinical variables, including COVID-19 severity, have been previously reported. However, almost all prior reports originated from retrospective cohorts in the USA and Europe; data from other global regions has been limited, and smaller longitudinal cohorts have spanned multiple variants, complicating data intepretations.

We observed comparatively shorter duration and lower rates of persistence among Peruvian participants and among non-Hispanic Black participants globally, and longer duration and higher rates of persistence in the USA compared to other regions, and among people with a history of lung disease, possibly due to higher pre-existing immunity to human coronaviruses reported in sub-Saharan Africa **(**SSA) than in USA [39]. Furthermore, non-Hispanic Black individuals were less likely than their non-Black counterparts to report symptoms for other illnesses, including stigmatising conditions like mental illness and HIV [40,41]; the potential stigma associated with a COVID-19 diagnosis during the study period (i.e. the first year of the pandemic), coupled with potentially limited health care access, socio-economic and other factors may have affected our observations of differential reports of symptom persistence by race.

The reporting of symptom duration and the spectrum of low (Peru) to high (USA) rates of symptom persistence reported by region may reflect similar cultural phenomena and highlight the unique limitations of characterizing post-COVID syndromes based almost entirely on data from the global North. The only country from the global North in this trial, the USA, was an outlier with nearly nine times higher odds of symptom persistence than Peruvians and nearly twice the odds of Africans, differences that were even more pronounced for some symptom classes; for example, those in the USA had over 20 times the odds of general symptoms and nearly 40 times the odds of neurologic symptoms compared to those in Peru. We observed these regional differences mostly in more qualitative (and more subjective) reports of symptom persistence beyond acute COVID-19 resolution, and less in more quantitative (and objective) reports of absolute symptom duration. These differences in reports of persistence may reflect cultural, social, or physiologic factors – COVID-19 was likely not more severe or persistent in the USA as in Peru or Africa, but different coping strategies, expectations of the timeline of acute COVID-19 resolution, access to therapeutic resources, or other psychosocial factors may have influenced these data. This variability highlights the importance of considering, controlling for, and carefully reporting regional differences in epidemiologic and clinical research outcomes and in the diagnosis and management of post-COVID syndromes.

Our observation of longer duration and higher rates of post-COVID symptoms among those with more severe disease are in line with other studies [12-14,25,31-33,35]. The persistence of symptoms in individuals who have recovered from more severe disease may reflect a longer period of virus exposure, a weaker or slower immune response to acute infection, and/or more protracted exposure to the inflammatory milieu of longer-lasting COVID-19. Interestingly, though invasive oxygen (i.e. mechanical ventilation) is associated with slower recovery and lingering cognitive and physical dysfunction from other illnesses [42], we did not observe a significant difference in post-COVID symptoms based on non-invasive vs invasive oxygen requirement. This may partially reflect the limitations of oxygen access during the first global COVID waves in 2020, when our trial was conducted, when invasive oxygen was in limited supply, even for individuals with severe illness; this phenomenon was particularly reported in Africa, where very few participants reported an invasive oxygen requirement.

We also observed a pronounced correlation of prolonged viral shedding and persistent gastrointestinal symptoms, consistent with a possible gastrointestinal viral reservoir, with persistent seeding of the nasopharynx from the gastrointestinal **(**GI) tract and vice versa. Future research may determine whether this observation might reflect unique immunologic responses, including alterations in mucosal immunoglobulin A (IgA) expression or cross-reactivity with SARS-CoV-2 and gut microbiome epitopes [43]. This finding may also highlight a potential therapeutic target (gastrointestinal health to prevent establishment of or to eradicate a possible viral reservoir) in patients with a chronic COVID-19 course characterised by GI symptoms that may be attributable to sustained viral replication and associated gastrointestinal inflammation [44,45]. Furthermore, our observation may support the hypothesis that children can be significant vectors of infection, considering early reports of more GI symptoms in children than in adults [46]. Lastly, with repeat testing to demonstrate viral clearance no longer required before cessation of isolation or masking recommendations, and definitions of COVID-19 resolution largely defined by time and respiratory (but not gastrointestinal) symptom trajectories, our data suggests that transmissions may be reduced by considering alternate testing or other guidance for individuals with gastrointestinal COVID-19 symptoms.

While the trial’s follow-up in six countries spanning the global north and south is a unique strength among post-COVID research, thus far, the global diversity regarding early testing and treatment resources and policies, and other influences on participants’ COVID-19 experience pose risks of selection bias and misinterpretation; several cultural and social factors could influence the results of regional comparisons and might be more important for less quantitative assessments of symptom “persistence” than for quantitative assessments of absolute symptom duration. We mitigated these risks by harmonising the eligibility assessment and other trial procedures, analytic approaches to control for potential confounders, and the stratification of the trial population with slots distributed by region. However, participant stratification by severity and age led to some smaller subgroups, which varied somewhat by region; some strata were more difficult to identify in some regions due to structural factors; for example, asymptomatic polymerase chain reaction (PCR) positive individuals were more difficult to identify in regions in which PCR testing was only easily accessible to individuals with symptoms (e.g. Peru). We were still able to detect differences within and across strata and regions by limiting this effect through analytic solutions, but these influences on the global data must be considered; larger subgroups in each region might identify additional robust post-COVID correlations. Finally, we enrolled individuals who had acquired SARS-CoV-2 in the first year of the pandemic, when ancestral Wuhan and early D614G-based variants were globally dominant and before vaccination was widely available. This facilitates comparability across the global cohort and limits confounding of potential variant- or vaccine-based influences on post-COVID clinical trajectories. However, it also may limit the applicability of post-COVID findings that could be influenced by viral variants and vaccination. Limited early data suggests that rates of post-COVID syndromes differ by variant [47], though how variant may interact with other correlations we identified is not known.

## CONCLUSIONS

As new variants emerge and COVID-19 cases multiply, persistent symptoms could have a high impact. Our data suggest that this impact is influenced by several factors, including both previously reported demographic and clinical variables and regional influences. We encourage additional research across diverse global settings to better understand the presentations and correlations of post-COVID syndrome globally. COVID-19 has been reported in essentially every country worldwide, but with varying impacts in different countries and regions. Understanding global differences and similarities can inform future research and management of COVID-19 and its sequelae worldwide.

## Additional material


Online Supplementary Document
