# Supplementary material for: Post-COVID symptom profiles and duration in a global convalescent COVID-19 observational cohort: Correlations with demographics, medical history, acute COVID-19 severity and global region
Source: J Glob Health. 2023 Jun 23;13:06020. doi: 10.7189/jogh.13.06020 (PMC10289480; doi:10.7189/jogh.13.06020)

Supplementary Material

HVTN 405/HPTN 1901 Study Team, Clinical Research Sites and Investigators of Record

Srilatha Edupuganti (Hope Clinic, Atlanta, GA); Valeria Cantos Lucio (Ponce de Leon, Atlanta, GA); Jason Farley (Johns Hopkins, Baltimore, MD); Paul A. Goepfert (Birmingham, AL); Lindsey R. Baden (Brigham and Womens Hospital, Boston, MA); Kenneth H. Mayer (Fenway Health, Boston, MA); Cynthia Gay (Chapel Hill, NC); Temitope Oyedele (AYAR at CORE, Chicago, IL); Juan Carlos Hinojosa Boyer (Asociacion Civil Selva Amazonica, Iquitos, Peru); Javier R. Lama (Barranco, Lima, Peru); Juan Jose Montenegro Idrogo (San Marcos/CITBM, Lima, Peru); Pedro Gonzales (San Miguel, Lima, Peru); Robinson Cabello (Via Libre, Lima, Peru); Raphael Landovitz (UCLA CARE Center, Los Angeles, CA); Spyros A. Kalams (Vanderbilt, Nashville, TN); Susan Abdalian (Adolescent Trials Unit, New Orleans, LA); Ellen Morrison (Bronx Prevention Center, New York, NY); Yael Hirsch-Moverman (Harlem Prevention Center, New York, NY); Hong Van Tieu (NY Blood Center, New York, NY); Magdalena Sobieszczyk (Physicians & Surgeons, New York, NY); Shobha Swaminathan (New Jersey Medical School, Newark, NJ); Ian Frank (Penn Prevention, Philadelphia, PA); Michael Keefer (University of Rochester, Rochester, NY); Susan P. Buchbinder (Bridge HIV, San Francisco, CA); M. Juliana McElrath (Seattle Vaccine Trials Unit, Seattle, WA); Manya Magnus (George Washington, Washington, DC); Terence Tafatatha (Lilongwe, Malawi); Zaheer Hoosain (Bloemfontein, South Africa); Gonasagrie Nair and Llewellyn Fleurs (Emavundleni, Cape Town, South Africa); Sheetal Kassim (Groote Schuur, Cape Town, South Africa); Amy Ward and Graeme Meintjes (Khayelitsha, Cape Town, South Africa); Elizabeth Spooner (Botha’s Hill and Tongaat, Durban, South Africa); Brodie Daniels (Chatsworth, Durban, South Africa); Nigel Garrett (eThekwini, Durban, South Africa); Brodie Daniels (Isipingo and Verulam, Durban, South Africa); Craig Innes (Klerksdorp, South Africa); Philip Kotze (Ladysmith, South Africa); Sheena Kotze (Mamelodi, South Africa); Katherine Gill (Masiphumelele, South Africa); Pamela Mda (Mthatha, South Africa); William Brumskine (Rustenburg, South Africa); Khatija Ahmed (Soshanguve, South Africa); Fatima Laher (Bara, Soweto, South Africa); Tricia Philip, Erica Lazarus and Anusha Nana (Kliptown, Soweto, South Africa); Modulakgotla Sebe (Clinic 3, Tembisa, South Africa); Kathryn Mngadi (Clinica 4, Tembisa, South Africa); Halima Dawood (Vulindlela, South Africa); Roma Chilengi and Katanekwa Njekwa (Matero, Lusaka, Zambia); Portia Hunidzarira and Nuchaneta Bhondai-Mhuri (Seke South, Harare, Zimbabwe); Portia Hunidzarira and Lynda Stranix-Chibanda (St. Mary’s, Harare, Zimbabwe); and Portia Hunidzarira and Nyaradzo Mgodi (Zengeza, Harare, Zimbabwe).

Tables

Supplemental Table 1. Eligibility criteria.

Age 18 or older
Reports having had a positive test for SARS-CoV-2
Reports resolution of COVID-19 within 1-8 weeks of enrollment or, if asymptomatic infection, reports positive SARS-CoV-2 test within 2-10 weeks of enrollment
Access to a participating HVTN or HPTN CRS and willingness to be followed for the planned duration of the study
Ability and willingness to provide informed consent
Assessment of understanding: volunteer demonstrates understanding of the study
Volunteers who were assigned female sex at birth and able to have children: negative pregnancy test within 4 days of enrollment visit

Supplemental Table 2. Trial schema.

**Group**		**N**
**1**	**Persons not hospitalized for COVID-19, without clinical spectrum or outcomes specified in group 3**	**439**
1A	Persons with asymptomatic infection, ages 18 through 55, inclusive	129
1B	Persons with asymptomatic infection, age >55	51
1C	Persons with symptomatic infection (i.e., COVID-19), ages 18 through 55	182
1D	Persons with symptomatic infection (i.e., COVID-19), age >55	77
**2**	**Persons previously hospitalized for COVID-19, without clinical spectrum or outcomes specified in group 3**	**196**
2A	Persons 18 through 55 years of age	122
2B	Persons >55 years of age	74
**3**	**Persons with specific clinical spectrums or outcomes, regardless of hospitalization history (e.g., persons recovered after intubation, with prolonged viral shedding, with myocarditis/pericarditis, with rapid recovery from COVID-19, with a second positive SARS-CoV-2 RT-PCR test result after a negative result)**	**125**

Supplemental Table 3. Clinical research site IRB name and number.

Site name	DAIDS ID	IRB name and no
Atlanta - Hope Clinic CRS	31440	Advarra SSU00120200
Atlanta - Ponce de Leon Center CRS	5802	Advarra SSU00120348
Baltimore – Johns Hopkins University CRS	201	Advarra SSU00121119
Birmingham	31788	Advarra SSU00121044
Boston – Brigham and Women’s Hospital Vaccine CRS	30007	Advarra SSU00120468
Boston - Fenway Health CRS	31785	Advarra SSU00120279
Chapel Hill	3201	Advarra SSU00120346
Chicago	31958	Advarra SSU00120225
Los Angeles – UCLA	601	Advarra SSU00120421
Nashville	30352	Advarra SSU00120455
New Orleans	31959	Advarra SSU00120321
New York – Bronx	30261	Advarra SSU00121076
New York – Harlem	30276	Advarra SSU00120537
New York - NYBC	31801	Advarra SSU00120370
New York – P&S	30329	Advarra SSU00120439
Newark	31786	Advarra SSU00120472
Philadelphia	30310	Advarra SSU00121269
Rochester	31467	Advarra SSU00121683
San Francisco - Bridge	30305	Advarra SSU00120245
Seattle	30331	Advarra SSU00120499
Washington DC	31608	Advarra SSU00120668
Lilongwe Malawi CR3025	12001	Ministry of health and population 2578
ACSA CRS	30259	Institution committee of bioethics 0052 -2020-CE
Lima - San Marcos/CITBM CRS	31970	Committee of Bioethics (CIB) 5591
Lima - Barranco	11301	Institution committee of bioethics 0050 -2020-CE
Lima - San Miguel CRS	11302	Institution committee of bioethics 0051 -2020-CE
Lima – Via Libre CRS	31909	Committee of Bioethics (CIB) 5592
Bloemfontein	32045	University of the Witwatersrand IRB 00001223
Cape Town - Emavundleni	30346	University of Cape Town HREC 345/2020
Cape Town – Groote Schuur	31708	University of Cape Town HREC 346/2020
Cape Town - Khayelitsha	31941	University of Cape Town HREC 344/2020
Durban - Botha’s Hill	31445	SAMRC Durban Westville EC024-7/20202
Durban - Chatsworth	30302	SAMRC Durban Westville EC024-7/20202
Durban - eThekwini	31422	BREC 00001464
Durban - Isipingo	31635	SAMRC Durban Westville EC024-7/20202

Durban - Tongaat	31662	SAMRC Durban Westville EC024-7/20202
Durban - Verulam	31663	SAMRC Durban Westville EC024-7/20202
Klerksdorp	30325	University of the Witwatersrand IRB 00001223
Ladysmith	31913	University of the Witwatersrand IRB 00001223
Mamelodi	31984	University of the Witwatersrand IRB 00001223
Masiphumelele	32023	University of Cape Town HREC 356/2020
Mthatha	31955	University of the Witwatersrand IRB 00001223
Rustenburg	31684	University of the Witwatersrand IRB 00001223
Soshanguve	31829	University of the Witwatersrand IRB 00001223
Soweto - Bara	30351	University of the Witwatersrand IRB 00001223
Soweto – Kliptown	31928	University of the Witwatersrand IRB 00001223
Tembisa – Clinic 3	31832	University of the Witwatersrand IRB 00001223
Tembisa – Clinic 4	32047	University of the Witwatersrand IRB 00001223
Vulindlela	31448	University of the Witwatersrand IRB 00001223
Lusaka- Matero	30290	UNZA BREC
Harare – Seke South	30294	JREC JREC/137/2020
Harare - St. Mary's	30303	JREC JREC/137/2020
Harare - Zengeza	30320	JREC JREC/137/2020

Supplemental Table 4. Associations of COVID-19 severity, medical co-morbidities, and demographics with persistence beyond acute COVID-19 resolution and duration of any symptoms among symptomatic participants by region.

Comparison[+]	OR	95% CI	P-Value	Q-Value[*]	GMR	95% CI	P-Value	Q-Value[*]
	**Persistence Beyond Acute COVID-19 Resolution**				**Symptom Duration**			
**A. Americas Cohort**								
COVID-19 Severity (Overall Test)	-	-	0.107	0.287	**-**	**-**	**<0.001**	**<0.001**
NIOR vs NOR	1.88	[1.04, 3.46]	0.037	0.287	**1.67**	**[1.36, 2.05]**	**<0.001**	**<0.001**
IOR vs NOR	1.62	[0.64, 4.06]	0.303	0.538	**1.71**	**[1.27, 2.31]**	**<0.001**	**0.002**
IOR vs NIOR	0.86	[0.33, 2.22]	0.76	0.936	1.03	[0.75, 1.4]	0.871	0.894
Age (>55 vs 18-55)	1.83	[1.07, 3.17]	0.029	0.287	**1.32**	**[1.09, 1.58]**	**0.004**	**0.013**
Sex at Birth (Male vs Female)	0.9	[0.52, 1.56]	0.708	0.936	0.88	[0.73, 1.06]	0.182	0.365
BMI (≥30 vs <30)	1.49	[0.87, 2.58]	0.146	0.33	**1.25**	**[1.04, 1.51]**	**0.02**	**0.054**
COPD/Emphysema/Asthma	2.1	[0.92, 5]	0.078	0.287	**1.54**	**[1.16, 2.06]**	**0.003**	**0.013**
Diabetes	1.07	[0.49, 2.32]	0.872	0.955	0.88	[0.67, 1.16]	0.363	0.528
Hypertension	0.88	[0.46, 1.67]	0.69	0.936	0.81	[0.65, 1.01]	0.06	0.137
HIV/AIDS	1.02	[0.44, 2.38]	0.955	0.955	1.07	[0.8, 1.44]	0.637	0.783
Prolonged Viral Shedding	2.02	[0.89, 4.69]	0.094	0.287	1.17	[0.87, 1.56]	0.299	0.479
Current Cigarettes/Marijuana Smoker	0.64	[0.25, 1.63]	0.345	0.552	0.98	[0.7, 1.37]	0.894	0.894
Ever Cigarettes/Marijuana Smoker	0.98	[0.56, 1.71]	0.943	0.955	1.02	[0.84, 1.24]	0.848	0.894
Non-Hispanic Black vs Non-His White	0.45	[0.18, 1.1]	0.079	0.287	0.89	[0.63, 1.26]	0.508	0.677
Others vs Non-His White	2.04	[0.76, 6.35]	0.165	0.33	1.21	[0.85, 1.73]	0.297	0.479
**B. Africa cohort**								
COVID-19 Severity (Overall Test)	-	-	0.055	0.207	-	-	0.05	0.25
NIOR vs NOR	1.59	[0.94, 2.72]	0.085	0.254	**1.33**	**[1.06, 1.67]**	**0.016**	**0.12**
IOR vs NOR	4.49	[1.15, 24.83]	0.03	0.207	1.22	[0.68, 2.18]	0.512	0.768
IOR vs NIOR	2.82	[0.7, 15.89]	0.151	0.294	0.92	[0.5, 1.67]	0.774	0.922
Age (>55 vs 18-55)	0.61	[0.33, 1.12]	0.112	0.28	0.89	[0.69, 1.15]	0.388	0.647
Sex at Birth (Male vs Female)	0.95	[0.57, 1.59]	0.845	0.905	0.94	[0.76, 1.17]	0.595	0.811
BMI (≥30 vs <30)	0.78	[0.46, 1.31]	0.348	0.48	0.97	[0.78, 1.22]	0.799	0.922
COPD/Emphysema/Asthma	1.3	[0.46, 3.71]	0.613	0.707	1.43	[0.91, 2.26]	0.122	0.366
Diabetes	1.3	[0.67, 2.51]	0.437	0.546	1.25	[0.95, 1.66]	0.117	0.366
Hypertension	1.55	[0.85, 2.86]	0.157	0.294	1.17	[0.9, 1.52]	0.234	0.501
HIV/AIDS	0.65	[0.3, 1.37]	0.258	0.429	0.99	[0.72, 1.36]	0.941	0.967
Prolonged Viral Shedding	2.44	[0.38, 25.95]	0.352	0.48	1.84	[0.77, 4.38]	0.17	0.426
Current Cigarettes/Marijuana Smoker	1.02	[0.41, 2.49]	0.957	0.957	0.83	[0.56, 1.22]	0.345	0.646
Ever Cigarettes/Marijuana Smoker	1.96	[1, 3.88]	0.05	0.207	0.99	[0.74, 1.33]	0.967	0.967
Others vs Non-His Black	**2.11**	**[1.23, 3.66]**	**0.006**	**0.094**	**1.37**	**[1.09, 1.72]**	**0.007**	**0.106**

[+]: Adjusted for a set of confounders (COVID-19 severity, age, sex assigned at birth, and region). For each of COVID-19 severity, age, sex assigned at birth, and region, the comparison was adjusted for the rest of confounders. Overall test for COVID-19 severity was done using Wald-test.

[*]: Adjusted for multiple comparisons to control false discovery rate for each cohort.

Bolded values for significant with p-value≤0.05 and q-value≤0.2.

NOR: symptomatic, no oxygen requirement; NIOR: symptomatic, non-invasive oxygen requirement; IOR: symptomatic, invasive oxygen requirement

Supplemental Table 5. Associations of COVID-19 Severity, medical co-morbidities, and demographics with persistence beyond acute COVID-19 resolution and duration of any symptom in each body system among all symptomatic participants.

		Persistence Beyond Acute COVID-19 Resolution				Symptom Duration			
**Body System**	**Comparison[+]**	**OR**	**95% CI**	**P-Value**	**Q-Value[*]**	**GMR**	**95% CI**	**P-Value**	**Q-Value[*]**
General	COVID-19 Severity (Overall Test)	**-**	**-**	**0.009**	**0.029**	**-**	**-**	**<0.001**	**<0.001**
	NIOR vs NOR	1.53	[0.98, 2.39]	0.064	0.134	**1.43**	**[1.22, 1.68]**	**<0.001**	**<0.001**
	IOR vs NOR	**3.38**	**[1.45, 8.01]**	**0.005**	**0.018**	**1.7**	**[1.28, 2.25]**	**<0.001**	**0.001**
	IOR vs NIOR	2.21	[0.93, 5.36]	0.073	0.14	1.19	[0.89, 1.59]	0.253	0.323
	Age (>55 vs 18-55)	1.32	[0.83, 2.07]	0.24	0.368	1.15	[0.98, 1.35]	0.096	0.158
	Sex at Birth (Male vs Female)	0.88	[0.57, 1.36]	0.571	0.773	0.89	[0.76, 1.03]	0.127	0.195
	BMI (≥30 vs <30)	0.92	[0.6, 1.42]	0.72	0.872	1.1	[0.94, 1.28]	0.241	0.323
	COPD/Emphysema/Asthma	**2.94**	**[1.48, 5.92]**	**0.002**	**0.01**	**1.58**	**[1.21, 2.06]**	**0.001**	**0.002**
	Diabetes	0.96	[0.54, 1.68]	0.887	0.956	0.94	[0.76, 1.17]	0.589	0.677
	Hypertension	1.03	[0.63, 1.66]	0.918	0.956	0.98	[0.82, 1.17]	0.826	0.864
	HIV/AIDS	1.02	[0.55, 1.84]	0.956	0.956	1.07	[0.86, 1.34]	0.532	0.644
	Prolonged Viral Shedding	1.97	[0.87, 4.42]	0.101	0.166	1.32	[0.99, 1.77]	0.063	0.121
	Current Cigarettes/Marijuana Smoker	1.14	[0.56, 2.27]	0.707	0.872	0.78	[0.6, 1.03]	0.076	0.134
	Ever Cigarettes/Marijuana Smoker	**1.71**	**[1.05, 2.8]**	**0.033**	**0.083**	1	[0.84, 1.19]	0.993	0.993
	Non-Hispanic Black vs Non-Hispanic White	**0.46**	**[0.22, 0.92]**	**0.027**	**0.079**	**0.72**	**[0.55, 0.95]**	**0.023**	**0.047**
	Others vs Non-His White	0.93	[0.46, 1.87]	0.835	0.956	1.05	[0.79, 1.4]	0.737	0.807
	Region (Overall Test)	**-**	**-**	**<0.001**	**<0.001**	**-**	**-**	**<0.001**	**<0.001**
	USA vs Peru	**20.77**	**[8.81, 59.63]**	**<0.001**	**<0.001**	**2.16**	**[1.77, 2.64]**	**<0.001**	**<0.001**
	Non-RSA vs RSA	0.79	[0.41, 1.48]	0.464	0.668	0.87	[0.68, 1.11]	0.25	0.323
	RSA vs Peru	**13.84**	**[5.81, 40.01]**	**<0.001**	**<0.001**	**1.56**	**[1.28, 1.9]**	**<0.001**	**<0.001**
	Non-RSA vs Peru	**10.91**	**[4.07, 33.87]**	**<0.001**	**<0.001**	**1.35**	**[1.05, 1.75]**	**0.022**	**0.047**
	RSA vs USA	0.67	[0.42, 1.07]	0.091	0.161	**0.72**	**[0.6, 0.87]**	**0.001**	**0.002**
	Non-RSA vs USA	**0.53**	**[0.27, 1]**	**0.049**	**0.113**	**0.63**	**[0.49, 0.81]**	**<0.001**	**0.001**
Central Nervous System (CNS)	COVID-19 Severity (Overall Test)	-	-	0.077	0.268	**-**	**-**	**<0.001**	**0.001**
	NIOR vs NOR	**1.73**	**[1.07, 2.79]**	**0.026**	**0.119**	**1.4**	**[1.17, 1.67]**	**<0.001**	**0.001**
	IOR vs NOR	0.99	[0.29, 2.84]	0.989	0.989	**1.53**	**[1.09, 2.15]**	**0.014**	**0.035**
	IOR vs NIOR	0.57	[0.16, 1.69]	0.324	0.479	1.1	[0.77, 1.57]	0.608	0.737

	Age (>55 vs 18-55)	1.24	[0.74, 2.04]	0.413	0.531	1.06	[0.89, 1.27]	0.509	0.651
	Sex at Birth (Male vs Female)	0.74	[0.46, 1.18]	0.212	0.479	**0.76**	**[0.65, 0.9]**	**0.002**	**0.005**
	BMI (≥30 vs <30)	0.89	[0.56, 1.42]	0.634	0.722	1.14	[0.97, 1.35]	0.122	0.255
	COPD/Emphysema/Asthma	1.43	[0.69, 2.9]	0.333	0.479	**1.57**	**[1.18, 2.08]**	**0.002**	**0.005**
	Diabetes	1.35	[0.73, 2.47]	0.332	0.479	1.13	[0.89, 1.43]	0.316	0.468
	Hypertension	1.34	[0.79, 2.26]	0.268	0.479	1	[0.82, 1.22]	0.998	0.998
	HIV/AIDS	0.66	[0.32, 1.28]	0.222	0.479	1.06	[0.83, 1.35]	0.66	0.759
	Prolonged Viral Shedding	1.54	[0.64, 3.52]	0.325	0.479	1.2	[0.87, 1.64]	0.27	0.444
	Current Cigarettes/Marijuana Smoker	0.85	[0.38, 1.82]	0.691	0.722	0.9	[0.67, 1.21]	0.481	0.65
	Ever Cigarettes/Marijuana Smoker	1.61	[0.94, 2.76]	0.081	0.268	1.02	[0.85, 1.24]	0.803	0.879
	Non-Hispanic Black vs Non-Hispanic White	0.52	[0.24, 1.12]	0.097	0.278	0.85	[0.62, 1.17]	0.326	0.468
	Others vs Non-His White	1.29	[0.61, 2.74]	0.501	0.606	1.2	[0.87, 1.65]	0.266	0.444
	Region (Overall Test)	**-**	**-**	**<0.001**	**0.001**	**-**	**-**	**<0.001**	**<0.001**
	USA vs Peru	**38.72**	**[10.09, 347.37]**	**<0.001**	**<0.001**	**1.79**	**[1.43, 2.23]**	**<0.001**	**<0.001**
	Non-RSA vs RSA	0.76	[0.38, 1.45]	0.415	0.531	1.18	[0.91, 1.54]	0.209	0.401
	RSA vs Peru	**34.65**	**[9.06, 310.52]**	**<0.001**	**<0.001**	**1.49**	**[1.2, 1.85]**	**<0.001**	**0.001**
	Non-RSA vs Peru	**26.41**	**[6.25, 245.41]**	**<0.001**	**<0.001**	**1.76**	**[1.33, 2.33]**	**<0.001**	**0.001**
	RSA vs USA	0.89	[0.54, 1.47]	0.662	0.722	0.83	[0.68, 1.03]	0.09	0.207
	Non-RSA vs USA	0.68	[0.34, 1.34]	0.268	0.479	0.99	[0.75, 1.3]	0.93	0.973
Gastrointestinal (GIT)	COVID-19 Severity (Overall Test)	-	-	0.327	0.502	-	-	0.066	0.253
	NIOR vs NOR	1.33	[0.67, 2.58]	0.405	0.547	**1.23**	**[1.01, 1.49]**	**0.041**	**0.187**
	IOR vs NOR	2.31	[0.65, 6.91]	0.18	0.298	1.32	[0.91, 1.92]	0.145	0.371
	IOR vs NIOR	1.73	[0.48, 5.39]	0.379	0.544	1.08	[0.73, 1.59]	0.706	0.921
	Age (>55 vs 18-55)	1.04	[0.52, 2.03]	0.913	0.928	1.02	[0.84, 1.24]	0.841	0.921
	Sex at Birth (Male vs Female)	**0.49**	**[0.24, 0.96]**	**0.038**	**0.18**	0.81	[0.68, 0.98]	0.03	0.171
	BMI (≥30 vs <30)	1.03	[0.54, 1.97]	0.928	0.928	1.15	[0.96, 1.39]	0.136	0.371
	COPD/Emphysema/Asthma	2.22	[0.92, 5.07]	0.074	0.213	1.12	[0.82, 1.54]	0.468	0.718
	Diabetes	0.86	[0.32, 2.03]	0.74	0.852	1.13	[0.87, 1.46]	0.367	0.703
	Hypertension	0.56	[0.24, 1.2]	0.14	0.277	1.03	[0.82, 1.28]	0.815	0.921
	HIV/AIDS	1.44	[0.56, 3.31]	0.428	0.547	1	[0.75, 1.33]	0.988	0.988
	Prolonged Viral Shedding	**4.52**	**[1.66, 11.78]**	**0.004**	**0.045**	1.24	[0.87, 1.78]	0.238	0.547
	Current Cigarettes/Marijuana Smoker	**0.12**	**[0, 0.92]**	**0.039**	**0.18**	0.84	[0.59, 1.19]	0.323	0.676
	Ever Cigarettes/Marijuana Smoker	0.54	[0.24, 1.14]	0.107	0.266	1.03	[0.84, 1.26]	0.776	0.921

	Non-Hispanic Black vs Non-Hispanic White	0.78	[0.26, 2.19]	0.641	0.776	0.95	[0.67, 1.36]	0.797	0.921
	Others vs Non-His White	0.44	[0.13, 1.31]	0.145	0.277	1.1	[0.77, 1.57]	0.605	0.87
	Region (Overall Test)	-	-	0.012	0.089	**-**	**-**	**0.016**	**0.171**
	USA vs Peru	**5.24**	**[1.99, 17.2]**	**<0.001**	**0.011**	**1.31**	**[1.03, 1.66]**	**0.026**	**0.171**
	Non-RSA vs RSA	0.91	[0.29, 2.48]	0.859	0.928	1.12	[0.83, 1.5]	0.457	0.718
	RSA vs Peru	2.67	[0.93, 9.2]	0.068	0.213	0.91	[0.71, 1.16]	0.453	0.718
	Non-RSA vs Peru	2.43	[0.65, 9.52]	0.181	0.298	1.02	[0.75, 1.38]	0.907	0.949
	RSA vs USA	0.51	[0.24, 1.06]	0.072	0.213	**0.69**	**[0.55, 0.88]**	**0.002**	**0.055**
	Non-RSA vs USA	0.46	[0.15, 1.2]	0.116	0.266	0.78	[0.58, 1.05]	0.096	0.315
Head, Eyes, Ears, Nose and Throat (HEENT)	COVID-19 Severity (Overall Test)	-	-	0.39	0.535	-	-	0.126	0.362
	NIOR vs NOR	1.53	[0.65, 3.44]	0.321	0.535	1.26	[1.01, 1.56]	0.043	0.306
	IOR vs NOR	2.4	[0.43, 9.61]	0.286	0.535	1.09	[0.72, 1.67]	0.673	0.845
	IOR vs NIOR	1.57	[0.28, 6.42]	0.573	0.599	0.87	[0.56, 1.35]	0.539	0.789
	Age (>55 vs 18-55)	0.68	[0.25, 1.62]	0.395	0.535	1.07	[0.86, 1.33]	0.541	0.789
	Sex at Birth (Male vs Female)	1.26	[0.58, 2.7]	0.557	0.599	1	[0.82, 1.22]	0.969	0.969
	BMI (≥30 vs <30)	0.5	[0.22, 1.11]	0.091	0.298	1.02	[0.83, 1.25]	0.836	0.915
	COPD/Emphysema/Asthma	1.33	[0.39, 3.78]	0.624	0.624	1.1	[0.8, 1.52]	0.549	0.789
	Diabetes	0.42	[0.08, 1.4]	0.172	0.361	1.08	[0.81, 1.43]	0.622	0.841
	Hypertension	0.33	[0.08, 0.98]	0.045	0.287	1.11	[0.86, 1.42]	0.428	0.757
	HIV/AIDS	0.58	[0.15, 1.67]	0.334	0.535	0.95	[0.72, 1.26]	0.73	0.845
	Prolonged Viral Shedding	3.36	[1.02, 9.88]	0.046	0.287	1.39	[0.97, 2]	0.071	0.306
	Current Cigarettes/Marijuana Smoker	2.89	[1, 7.84]	0.05	0.287	1.19	[0.84, 1.68]	0.33	0.691
	Ever Cigarettes/Marijuana Smoker	2.16	[0.93, 5.05]	0.072	0.298	1.24	[1, 1.55]	0.055	0.306
	Non-Hispanic Black vs Non-Hispanic White	0.32	[0.07, 1.3]	0.112	0.298	1.07	[0.72, 1.59]	0.734	0.845
	Others vs Non-His White	0.65	[0.16, 2.43]	0.533	0.599	1.25	[0.85, 1.85]	0.256	0.59
	Region (Overall Test)	-	-	0.117	0.298	-	-	0.033	0.306
	USA vs Peru	2.49	[0.81, 8.83]	0.113	0.298	**1.47**	**[1.13, 1.9]**	**0.004**	**0.087**
	Non-RSA vs RSA	0.44	[0.11, 1.34]	0.154	0.355	1.02	[0.75, 1.38]	0.898	0.939
	RSA vs Peru	3.73	[1.28, 13.17]	0.015	0.287	1.26	[0.97, 1.63]	0.08	0.306
	Non-RSA vs Peru	1.63	[0.35, 7.03]	0.517	0.599	1.29	[0.94, 1.75]	0.115	0.362
	RSA vs USA	1.5	[0.63, 3.78]	0.367	0.535	0.86	[0.66, 1.11]	0.241	0.59
	Non-RSA vs USA	0.65	[0.16, 2.18]	0.503	0.599	0.88	[0.64, 1.2]	0.402	0.757
Respiratory	COVID-19 Severity (Overall Test)	-	-	**0.03**	**0.113**	**-**	**-**	**<0.001**	**<0.001**

	NIOR vs NOR	**1.74**	**[1.15, 2.64]**	**0.009**	**0.04**	**1.6**	**[1.35, 1.9]**	**<0.001**	**<0.001**
	IOR vs NOR	1.52	[0.7, 3.14]	0.279	0.559	**1.58**	**[1.17, 2.13]**	**0.003**	**0.013**
	IOR vs NIOR	0.87	[0.4, 1.83]	0.719	0.889	0.99	[0.72, 1.34]	0.933	0.962
	Age (>55 vs 18-55)	0.95	[0.61, 1.45]	0.806	0.889	1.04	[0.88, 1.24]	0.643	0.846
	Sex at Birth (Male vs Female)	1.05	[0.7, 1.57]	0.812	0.889	0.97	[0.82, 1.14]	0.699	0.846
	BMI (≥30 vs <30)	1.37	[0.92, 2.06]	0.124	0.357	**1.19**	**[1.01, 1.4]**	**0.036**	**0.076**
	COPD/Emphysema/Asthma	1.54	[0.8, 2.89]	0.193	0.463	**1.45**	**[1.1, 1.9]**	**0.008**	**0.025**
	Diabetes	0.82	[0.46, 1.41]	0.468	0.769	1.16	[0.93, 1.46]	0.189	0.335
	Hypertension	0.9	[0.56, 1.42]	0.639	0.865	0.89	[0.73, 1.07]	0.215	0.353
	HIV/AIDS	0.68	[0.36, 1.23]	0.201	0.463	1.02	[0.8, 1.3]	0.88	0.962
	Prolonged Viral Shedding	1.55	[0.68, 3.4]	0.292	0.559	1.04	[0.74, 1.47]	0.806	0.927
	Current Cigarettes/Marijuana Smoker	0.97	[0.46, 1.96]	0.943	0.946	1.13	[0.84, 1.53]	0.409	0.627
	Ever Cigarettes/Marijuana Smoker	1.02	[0.63, 1.62]	0.946	0.946	0.96	[0.8, 1.16]	0.669	0.846
	Non-Hispanic Black vs Non-Hispanic White	**0.36**	**[0.17, 0.76]**	**0.007**	**0.04**	**0.67**	**[0.49, 0.92]**	**0.013**	**0.034**
	Others vs Non-His White	0.8	[0.37, 1.69]	0.561	0.817	0.77	[0.56, 1.06]	0.107	0.205
	Region (Overall Test)	**-**	**-**	**0.007**	**0.04**	**-**	**-**	**0.001**	**0.003**
	USA vs Peru	**2.65**	**[1.51, 4.76]**	**0.001**	**0.014**	**1.34**	**[1.08, 1.67]**	**0.009**	**0.025**
	Non-RSA vs RSA	0.9	[0.48, 1.66]	0.736	0.889	**1.45**	**[1.12, 1.88]**	**0.005**	**0.018**
	RSA vs Peru	**2.31**	**[1.32, 4.12]**	**0.003**	**0.035**	0.93	[0.75, 1.15]	0.493	0.709
	Non-RSA vs Peru	**2.07**	**[1.02, 4.2]**	**0.044**	**0.146**	**1.35**	**[1.03, 1.78]**	**0.033**	**0.076**
	RSA vs USA	0.87	[0.54, 1.41]	0.569	0.817	**0.69**	**[0.56, 0.85]**	**<0.001**	**0.003**
	Non-RSA vs USA	0.78	[0.4, 1.48]	0.452	0.769	1.01	[0.77, 1.32]	0.962	0.962
Skin	COVID-19 Severity (Overall Test)	-	-	0.303	0.889	-	-	-	-
	NIOR vs NOR	5.47	[0.85, 63.19]	0.074	0.855	-	-	-	-
	IOR vs NOR	2.06	[0.14, 28.09]	0.578	0.889	-	-	-	-
	IOR vs NIOR	0.38	[0.02, 4.81]	0.459	0.889	-	-	-	-
	Age (>55 vs 18-55)	0.61	[0.09, 3.42]	0.574	0.889	-	-	-	-
	Sex at Birth (Male vs Female)	0.19	[0.02, 1.08]	0.061	0.855	-	-	-	-
	BMI (≥30 vs <30)	1.91	[0.45, 9.05]	0.38	0.889	-	-	-	-
	COPD/Emphysema/Asthma	0.53	[0.06, 3.48]	0.515	0.889	-	-	-	-
	Diabetes	1.65	[0.18, 13.52]	0.639	0.889	-	-	-	-
	Hypertension	2.86	[0.54, 21.76]	0.224	0.889	-	-	-	-
	HIV/AIDS	0.94	[0.08, 7.33]	0.951	0.951	-	-	-	-
	Prolonged Viral Shedding	2.41	[0.35, 21.79]	0.369	0.889	-	-	-	-
	Current Cigarettes/Marijuana Smoker	0.65	[0.05, 5.83]	0.699	0.889	-	-	-	-

Ever Cigarettes/Marijuana Smoker	0.93	[0.17, 5.01]	0.936	0.951	-	-	-	-
Non-Hispanic Black vs Non-Hispanic White	0.32	[0.01, 5.09]	0.432	0.889	-	-	-	-
Others vs Non-His White	0.64	[0.07, 4.41]	0.655	0.889	-	-	-	-
Region (Overall Test)	-	-	0.672	0.889	-	-	-	-
USA vs Peru	4.19	[0.26, 631.39]	0.339	0.889	-	-	-	-
Non-RSA vs RSA	1.13	[0.09, 12.94]	0.922	0.951	-	-	-	-
RSA vs Peru	1.57	[0.07, 260.57]	0.793	0.912	-	-	-	-
Non-RSA vs Peru	1.77	[0.08, 284.56]	0.734	0.889	-	-	-	-
RSA vs USA	0.37	[0.05, 2.46]	0.309	0.889	-	-	-	-
Non-RSA vs USA	0.42	[0.04, 2.6]	0.366	0.889	-	-	-	-

[+]: Adjusted for a set of confounders (COVID-19 severity, age, sex assigned at birth, and region). For each of COVID-19 severity, age, sex assigned at birth, and region, the comparison was adjusted for the rest of confounders. Overall test for COVID-19 severity and region was done using Wald-test.

[*]: Adjusted for multiple comparisons to control false discovery rate for each body system.

Bolded values for significant with p-value≤0.05 and q-value≤0.2.

NOR: symptomatic, no oxygen requirement; NIOR: symptomatic, non-invasive oxygen requirement; IOR: symptomatic, invasive oxygen requirement

Supplemental Table 6. Use of concomitant medications.

Medications category	Sub category		Total (n=759)	Peru (n=191)	USA (n=197)	RSA (n=286)	Non-RSA (n=85)	p-value
Any	Any	N	303 (39.9%)	32 (16.8%)	96 (48.7%)	161 (56.3%)	14 (16.5%)	<0.001
		Y	456 (60.1%)	159 (83.2%)	101 (51.3%)	125 (43.7%)	71 (83.5%)	
Anticoagulants	Any	N	693 (91.3%)	156 (81.7%)	190 (96.4%)	276 (96.5%)	71 (83.5%)	<0.001
		Y	66 (8.7%)	35 (18.3%)	7 (3.6%)	10 (3.5%)	14 (16.5%)	
	Enoxaparin	N	697 (91.8%)	157 (82.2%)	191 (97%)	277 (96.9%)	72 (84.7%)	<0.001
		Y	62 (8.2%)	34 (17.8%)	6 (3%)	9 (3.1%)	13 (15.3%)	
	Other anticoagulants	N	751 (98.9%)	189 (99%)	195 (99%)	284 (99.3%)	83 (97.6%)	0.787
		Y	8 (1.1%)	2 (1%)	2 (1%)	2 (0.7%)	2 (2.4%)	
Anti-infectives	Any	N	471 (62.1%)	50 (26.2%)	144 (73.1%)	241 (84.3%)	36 (42.4%)	<0.001
		Y	288 (37.9%)	141 (73.8%)	53 (26.9%)	45 (15.7%)	49 (57.6%)	
	Antifungals	N	755 (99.5%)	188 (98.4%)	197 (100%)	285 (99.7%)	85 (100%)	0.228
		Y	4 (0.5%)	3 (1.6%)	-	1 (0.3%)	-	
	Azithromycin	N	544 (71.7%)	68 (35.6%)	163 (82.7%)	265 (92.7%)	48 (56.5%)	<0.001
		Y	215 (28.3%)	123 (64.4%)	34 (17.3%)	21 (7.3%)	37 (43.5%)	
	Chloroquine/hydroxychloroquine	N	637 (83.9%)	115 (60.2%)	168 (85.3%)	280 (97.9%)	74 (87.1%)	<0.001
		Y	122 (16.1%)	76 (39.8%)	29 (14.7%)	6 (2.1%)	11 (12.9%)	
	Ivermectin	N	699 (92.1%)	131 (68.6%)	197 (100%)	286 (100%)	85 (100%)	<0.001
		Y	60 (7.9%)	60 (31.4%)	-	-	-	
	Oral antiseptics	N	754 (99.3%)	191 (100%)	197 (100%)	281 (98.3%)	85 (100%)	0.080
		Y	5 (0.7%)	-	-	5 (1.7%)	-	
	Other antibiotics	N	655 (86.3%)	153 (80.1%)	182 (92.4%)	264 (92.3%)	56 (65.9%)	<0.001
		Y	104 (13.7%)	38 (19.9%)	15 (7.6%)	22 (7.7%)	29 (34.1%)	
	Other antivirals	N	756 (99.6%)	191 (100%)	195 (99%)	285 (99.7%)	85 (100%)	0.552
		Y	3 (0.4%)	-	2 (1%)	1 (0.3%)	-	
	Remdesivir	N	749 (98.7%)	191 (100%)	190 (96.4%)	284 (99.3%)	84 (98.8%)	0.027
		Y	10 (1.3%)	-	7 (3.6%)	2 (0.7%)	1 (1.2%)	
Covid-19 vaccines	Any	N	707 (93.1%)	183 (95.8%)	153 (77.7%)	286 (100%)	85 (100%)	<0.001
		Y	52 (6.9%)	8 (4.2%)	44 (22.3%)	-	-	
Immunomodulators	Any	N	641 (84.5%)	131 (68.6%)	192 (97.5%)	250 (87.4%)	68 (80%)	<0.001
		Y	118 (15.5%)	60 (31.4%)	5 (2.5%)	36 (12.6%)	17 (20%)	
	Dexamethasone	N	720 (94.9%)	162 (84.8%)	197 (100%)	284 (99.3%)	77 (90.6%)	<0.001
		Y	39 (5.1%)	29 (15.2%)	-	2 (0.7%)	8 (9.4%)	
	Other corticosteroids	N	670 (88.3%)	149 (78%)	195 (99%)	251 (87.8%)	75 (88.2%)	<0.001

		Y	89 (11.7%)	42 (22%)	2 (1%)	35 (12.2%)	10 (11.8%)	
	Tocilizumab	N	749 (98.7%)	184 (96.3%)	194 (98.5%)	286 (100%)	85 (100%)	0.011
		Y	10 (1.3%)	7 (3.7%)	3 (1.5%)	-	-	
Symptomatic relief	Any	N	517 (68.1%)	114 (59.7%)	154 (78.2%)	210 (73.4%)	39 (45.9%)	<0.001
		Y	242 (31.9%)	77 (40.3%)	43 (21.8%)	76 (26.6%)	46 (54.1%)	
	Antiacids/antiemetics/antidiarrheals	N	734 (96.7%)	176 (92.1%)	193 (98%)	281 (98.3%)	84 (98.8%)	0.002
		Y	25 (3.3%)	15 (7.9%)	4 (2%)	5 (1.7%)	1 (1.2%)	
	Antihistamines/decongestants	N	729 (96%)	183 (95.8%)	194 (98.5%)	276 (96.5%)	76 (89.4%)	0.011
		Y	30 (4%)	8 (4.2%)	3 (1.5%)	10 (3.5%)	9 (10.6%)	
	Bronchodilators	N	731 (96.3%)	181 (94.8%)	186 (94.4%)	284 (99.3%)	80 (94.1%)	0.020
		Y	28 (3.7%)	10 (5.2%)	11 (5.6%)	2 (0.7%)	5 (5.9%)	
	Expectorants/antitussives	N	701 (92.4%)	177 (92.7%)	191 (97%)	256 (89.5%)	77 (90.6%)	0.048
		Y	58 (7.6%)	14 (7.3%)	6 (3%)	30 (10.5%)	8 (9.4%)	
	Fluids and electrolytes	N	749 (98.7%)	189 (99%)	195 (99%)	286 (100%)	79 (92.9%)	<0.001
		Y	10 (1.3%)	2 (1%)	2 (1%)	-	6 (7.1%)	
	Nsaids	N	708 (93.3%)	172 (90.1%)	193 (98%)	269 (94.1%)	74 (87.1%)	0.004
		Y	51 (6.7%)	19 (9.9%)	4 (2%)	17 (5.9%)	11 (12.9%)	
	Opiods	N	756 (99.6%)	191 (100%)	197 (100%)	283 (99%)	85 (100%)	0.289
		Y	3 (0.4%)	-	-	3 (1%)	-	
	Other analgesics	N	753 (99.2%)	191 (100%)	197 (100%)	282 (98.6%)	83 (97.6%)	0.131
		Y	6 (0.8%)	-	-	4 (1.4%)	2 (2.4%)	
	Paracetamol	N	574 (75.6%)	130 (68.1%)	157 (79.7%)	241 (84.3%)	46 (54.1%)	<0.001
		Y	185 (24.4%)	61 (31.9%)	40 (20.3%)	45 (15.7%)	39 (45.9%)	
Vitamins/supplements/herbals	Any	N	628 (82.7%)	191 (100%)	194 (98.5%)	218 (76.2%)	25 (29.4%)	<0.001
		Y	131 (17.3%)	-	3 (1.5%)	68 (23.8%)	60 (70.6%)	
	Ascorbic acid	N	700 (92.2%)	191 (100%)	196 (99.5%)	253 (88.5%)	60 (70.6%)	<0.001
		Y	59 (7.8%)	-	1 (0.5%)	33 (11.5%)	25 (29.4%)	
	Herbals and natural preparations	N	690 (90.9%)	191 (100%)	195 (99%)	264 (92.3%)	40 (47.1%)	<0.001
		Y	69 (9.1%)	-	2 (1%)	22 (7.7%)	45 (52.9%)	
	Other vitamins/supplements	N	726 (95.7%)	191 (100%)	197 (100%)	259 (90.6%)	79 (92.9%)	<0.001
		Y	33 (4.3%)	-	-	27 (9.4%)	6 (7.1%)	
	Zinc	N	727 (95.8%)	191 (100%)	196 (99.5%)	274 (95.8%)	66 (77.6%)	<0.001
		Y	32 (4.2%)	-	1 (0.5%)	12 (4.2%)	19 (22.4%)	
Other drugs	Any	N	712 (93.8%)	178 (93.2%)	186 (94.4%)	273 (95.5%)	75 (88.2%)	0.190
		Y	47 (6.2%)	13 (6.8%)	11 (5.6%)	13 (4.5%)	10 (11.8%)	

Supplemental Table 7. Association of concomitant medications (Yes/No) with symptom duration after adjusting for COVID-19 severity, age, sex assigned at birth, and region.

Body System	Comparison	Sample Size	GMR	95% CI	P-Value	Q-Value*
**Any Concomitant medications**						
Any	Yes vs. No	410 vs. 167	**1.33**	**[1.14, 1.56]**	**<0.001**	**0.003**
General	Yes vs. No	380 vs. 146	**1.28**	**[1.08, 1.52]**	**0.005**	**0.016**
GIT	Yes vs. No	287 vs. 109	**1.27**	**[1.03, 1.57]**	**0.024**	**0.033**
CNS	Yes vs. No	359 vs. 136	**1.25**	**[1.04, 1.51]**	**0.019**	**0.033**
HEENT	Yes vs. No	268 vs. 78	1.2	[0.94, 1.52]	0.139	0.162
Respiratory	Yes vs. No	368 vs. 142	**1.26**	**[1.06, 1.52]**	**0.011**	**0.026**
Skin	Yes vs. No	30 vs. 8	1.12	[0.42, 2.98]	0.821	0.821
**Corticosteroids**						
Any	Yes vs. No	86 vs. 491	1.24	[1.02, 1.52]	0.035	0.211
General	Yes vs. No	83 vs. 443	1.18	[0.96, 1.46]	0.122	0.244
GIT	Yes vs. No	54 vs. 342	1.17	[0.89, 1.53]	0.268	0.322
CNS	Yes vs. No	74 vs. 421	1.1	[0.87, 1.39]	0.414	0.414
HEENT	Yes vs. No	60 vs. 286	1.19	[0.92, 1.55]	0.186	0.279
Respiratory	Yes vs. No	83 vs. 427	1.22	[0.97, 1.52]	0.085	0.244
**Anticoagulants**						
Any	Yes vs. No	65 vs. 512	1.12	[0.89, 1.41]	0.343	0.8
General	Yes vs. No	63 vs. 463	1.2	[0.95, 1.53]	0.131	0.585
GIT	Yes vs. No	49 vs. 347	0.97	[0.73, 1.3]	0.85	0.921
CNS	Yes vs. No	57 vs. 438	1.21	[0.92, 1.59]	0.167	0.585
HEENT	Yes vs. No	40 vs. 306	0.98	[0.72, 1.35]	0.921	0.921
Respiratory	Yes vs. No	63 vs. 447	1.1	[0.85, 1.42]	0.469	0.821
Skin	Yes vs. No	6 vs. 32	0.7	[0.18, 2.82]	0.625	0.875
**Anti-infectives**						
Any	Yes vs. No	264 vs. 313	**1.4**	**[1.2, 1.63]**	**<0.001**	**<0.001**
General	Yes vs. No	250 vs. 276	**1.27**	**[1.07, 1.5]**	**0.006**	**0.009**
GIT	Yes vs. No	190 vs. 206	**1.47**	**[1.19, 1.8]**	**<0.001**	**0.001**
CNS	Yes vs. No	229 vs. 266	**1.43**	**[1.19, 1.72]**	**<0.001**	**<0.001**
HEENT	Yes vs. No	174 vs. 172	**1.27**	**[1.03, 1.58]**	**0.027**	**0.031**
Respiratory	Yes vs. No	241 vs. 269	**1.43**	**[1.2, 1.7]**	**<0.001**	**<0.001**
Skin	Yes vs. No	18 vs. 20	0.96	[0.39, 2.35]	0.934	0.934
**Immunomodulators**						
Any	Yes vs. No	115 vs. 462	**1.3**	**[1.08, 1.57]**	**0.006**	**0.042**

General	Yes vs. No	110 vs. 416	1.21	[0.99, 1.47]	0.057	0.133
GIT	Yes vs. No	72 vs. 324	1.16	[0.9, 1.49]	0.258	0.301
CNS	Yes vs. No	101 vs. 394	1.18	[0.95, 1.46]	0.144	0.202
HEENT	Yes vs. No	79 vs. 267	1.25	[0.98, 1.61]	0.078	0.136
Respiratory	Yes vs. No	112 vs. 398	**1.28**	**[1.04, 1.57]**	**0.019**	**0.068**
Skin	Yes vs. No	6 vs. 32	1.67	[0.41, 6.76]	0.481	0.481
**Other Drugs**						
Any	Yes vs. No	47 vs. 530	1.02	[0.79, 1.32]	0.871	0.871
General	Yes vs. No	43 vs. 483	1.11	[0.84, 1.46]	0.457	0.871
GIT	Yes vs. No	40 vs. 356	0.84	[0.62, 1.14]	0.272	0.871
CNS	Yes vs. No	41 vs. 454	1.16	[0.86, 1.57]	0.335	0.871
HEENT	Yes vs. No	28 vs. 318	1.04	[0.72, 1.51]	0.822	0.871
Respiratory	Yes vs. No	44 vs. 466	0.95	[0.71, 1.26]	0.707	0.871
**Symptomatic Relief**						
Any	Yes vs. No	237 vs. 340	**1.26**	**[1.1, 1.46]**	**0.001**	**0.006**
General	Yes vs. No	222 vs. 304	**1.27**	**[1.1, 1.48]**	**0.002**	**0.006**
GIT	Yes vs. No	167 vs. 229	**1.23**	**[1.02, 1.48]**	**0.029**	**0.05**
CNS	Yes vs. No	213 vs. 282	**1.19**	**[1.01, 1.41]**	**0.037**	**0.052**
HEENT	Yes vs. No	159 vs. 187	**1.35**	**[1.11, 1.64]**	**0.003**	**0.006**
Respiratory	Yes vs. No	224 vs. 286	1.14	[0.97, 1.34]	0.124	0.144
Skin	Yes vs. No	17 vs. 21	0.75	[0.32, 1.73]	0.505	0.505
**Vitamins/Supplements/Herbals**						
Any	Yes vs. No	114 vs. 463	**1.38**	**[1.12, 1.69]**	**0.003**	**0.019**
General	Yes vs. No	104 vs. 422	1.19	[0.95, 1.49]	0.13	0.304
GIT	Yes vs. No	82 vs. 314	1.2	[0.91, 1.57]	0.199	0.349
CNS	Yes vs. No	108 vs. 387	1.12	[0.88, 1.42]	0.346	0.48
HEENT	Yes vs. No	74 vs. 272	1.13	[0.85, 1.5]	0.412	0.48
Respiratory	Yes vs. No	101 vs. 409	**1.36**	**[1.07, 1.72]**	**0.011**	**0.039**
Skin	Yes vs. No	9 vs. 29	0.94	[0.34, 2.59]	0.91	0.91

Supplemental Table 8. Summary of persistence beyond acute COVID-19 resolution and symptom duration by region.

Body System	Symptom	Outcome	Statistics	Total	Peru	USA	RSA	Non-RSA	p-value
Any System	Any Symptom	Symptom	N (%)	578 (100%)	150 (100%)	152 (100%)	209 (100%)	67 (100%)	-
		Persistence	N (%)	251 (43%)	26 (17.3%)	98 (64.5%)	95 (45.5%)	32 (47.8%)	<0.001
		Duration (in Days)	Median (IQR)	20 (11, 35)	16 (10, 24)	30 (16, 46.5)	17 (8, 30)	25 (14, 42.5)	<0.001
		Duration Days >30	N (%)	163 (28%)	23 (15.3%)	69 (45.4%)	47 (22.5%)	24 (35.8%)	-
		Duration Days >60	N (%)	46 (8%)	2 (1.3%)	24 (15.8%)	12 (5.7%)	8 (11.9%)	-
Central Nervous System (CNS)	Any	Symptom	N (%)	496 (86%)	123 (82%)	133 (87.5%)	178 (85.2%)	62 (92.5%)	0.195
		Persistence	N (%)	114 (23%)	1 (0.8%)	44 (33.1%)	54 (30.3%)	15 (24.2%)	<0.001
		Duration (in Days)	Median (IQR)	11 (7, 21)	8 (5, 13.5)	14 (7, 30)	12 (7, 21)	14 (7, 30)	<0.001
		Duration Days >30	N (%)	71 (14%)	2 (1.6%)	30 (22.6%)	24 (13.5%)	15 (24.2%)	-
		Duration Days >60	N (%)	23 (5%)	0 (0%)	12 (9%)	7 (3.9%)	4 (6.5%)	-
	Altered mental status/confusion	Symptom	N (%)	94 (16%)	8 (5.3%)	43 (28.3%)	24 (11.5%)	19 (28.4%)	<0.001
		Persistence	N (%)	18 (19%)	0 (0%)	10 (23.3%)	5 (20.8%)	3 (15.8%)	0.467
		Duration (in Days)	Median (IQR)	7 (3, 14)	7.5 (2.8, 10)	7 (4, 21)	10 (2.8, 17.2)	3 (3, 7)	0.235
		Duration Days >30	N (%)	12 (13%)	0 (0%)	7 (16.3%)	3 (12.5%)	2 (10.5%)	-
		Duration Days >60	N (%)	7 (7%)	0 (0%)	4 (9.3%)	3 (12.5%)	0 (0%)	-
	Anosmia/Hyposmia	Symptom	N (%)	314 (54%)	71 (47.3%)	87 (57.2%)	112 (53.6%)	44 (65.7%)	0.072
		Persistence	N (%)	49 (16%)	0 (0%)	24 (27.6%)	18 (16.1%)	7 (15.9%)	<0.001
		Duration (in Days)	Median (IQR)	10 (6, 18)	7 (4, 10)	14 (7, 30)	10 (6, 15.2)	14 (7, 21.8)	<0.001
		Duration Days >30	N (%)	33 (11%)	1 (1.4%)	17 (19.5%)	9 (8%)	6 (13.6%)	-
		Duration Days >60	N (%)	7 (2%)	0 (0%)	5 (5.7%)	1 (0.9%)	1 (2.3%)	-
	Headache	Symptom	N (%)	374 (65%)	87 (58%)	105 (69.1%)	134 (64.1%)	48 (71.6%)	0.129
		Persistence	N (%)	63 (17%)	1 (1.1%)	19 (18.1%)	36 (26.9%)	7 (14.6%)	<0.001
		Duration (in Days)	Median (IQR)	8 (4, 14)	7 (4, 10)	10 (3, 14)	8 (4, 15)	7 (4, 21.2)	0.261
		Duration Days >30	N (%)	36 (10%)	1 (1.1%)	13 (12.4%)	13 (9.7%)	9 (18.8%)	-
		Duration Days >60	N (%)	12 (3%)	0 (0%)	5 (4.8%)	4 (3%)	3 (6.2%)	-
	Hypogeusia	Symptom	N (%)	351 (61%)	82 (54.7%)	90 (59.2%)	125 (59.8%)	54 (80.6%)	0.003
		Persistence	N (%)	47 (13%)	0 (0%)	22 (24.4%)	18 (14.4%)	7 (13%)	<0.001
		Duration (in Days)	Median (IQR)	10 (6, 17.2)	7 (4, 10)	14 (7, 23.5)	10 (6, 15)	14 (7, 18.8)	<0.001
		Duration Days >30	N (%)	27 (8%)	1 (1.2%)	15 (16.7%)	9 (7.2%)	2 (3.7%)	-
		Duration Days >60	N (%)	4 (1%)	0 (0%)	3 (3.3%)	1 (0.8%)	0 (0%)	-
Gastrointestinal (GIT)	Any	Symptom	N (%)	397 (69%)	104 (69.3%)	119 (78.3%)	123 (58.9%)	51 (76.1%)	<0.001
		Persistence	N (%)	46 (12%)	4 (3.8%)	23 (19.3%)	14 (11.4%)	5 (9.8%)	0.004
		Duration (in Days)	Median (IQR)	7.5 (4, 14)	7 (5, 12)	10 (5, 19.8)	7 (4, 14)	7 (4, 12)	0.019
		Duration Days >30	N (%)	23 (6%)	3 (2.9%)	11 (9.2%)	4 (3.3%)	5 (9.8%)	-
		Duration Days >60	N (%)	6 (2%)	1 (1%)	5 (4.2%)	0 (0%)	0 (0%)	-
	Abdominal pain	Symptom	N (%)	99 (17%)	27 (18%)	25 (16.4%)	30 (14.4%)	17 (25.4%)	0.215
		Persistence	N (%)	10 (10%)	2 (7.4%)	5 (20%)	3 (10%)	0 (0%)	0.185
		Duration (in Days)	Median (IQR)	5 (3, 10.5)	5 (3, 7)	7 (3, 14)	7 (3, 14)	5 (3, 7)	0.330
		Duration Days >30	N (%)	3 (3%)	1 (3.7%)	2 (8%)	0 (0%)	0 (0%)	-

		Duration Days >60	N (%)	2 (2%)	0 (0%)	2 (8%)	0 (0%)	0 (0%)	-
	Anorexia	Symptom	N (%)	270 (47%)	67 (44.7%)	86 (56.6%)	80 (38.3%)	37 (55.2%)	0.003
		Persistence	N (%)	16 (6%)	2 (3%)	6 (7%)	4 (5%)	4 (10.8%)	0.405
		Duration (in Days)	Median (IQR)	10 (6, 14)	8 (5, 12)	10 (7, 17)	8 (5, 14)	9 (7, 14)	0.118
		Duration Days >30	N (%)	16 (6%)	2 (3%)	6 (7%)	3 (3.8%)	5 (13.5%)	-
		Duration Days >60	N (%)	2 (1%)	1 (1.5%)	1 (1.2%)	0 (0%)	0 (0%)	-
	Diarrhea	Symptom	N (%)	229 (40%)	73 (48.7%)	85 (55.9%)	45 (21.5%)	26 (38.8%)	<0.001
		Persistence	N (%)	20 (9%)	0 (0%)	14 (16.5%)	5 (11.1%)	1 (3.8%)	0.002
		Duration (in Days)	Median (IQR)	5 (2, 8)	4 (3, 7)	6 (3, 14)	4 (2, 7)	3 (2, 7)	0.034
		Duration Days >30	N (%)	6 (3%)	0 (0%)	6 (7.1%)	0 (0%)	0 (0%)	-
		Duration Days >60	N (%)	3 (1%)	0 (0%)	3 (3.5%)	0 (0%)	0 (0%)	-
	Nausea/Vomiting	Symptom	N (%)	161 (28%)	37 (24.7%)	46 (30.3%)	51 (24.4%)	27 (40.3%)	0.055
		Persistence	N (%)	10 (6%)	0 (0%)	5 (10.9%)	4 (7.8%)	1 (3.7%)	0.196
		Duration (in Days)	Median (IQR)	5 (2, 9)	3 (2, 7)	6.5 (2.2, 13)	5 (2.5, 7.5)	3 (1.5, 7)	0.242
		Duration Days >30	N (%)	4 (2%)	0 (0%)	2 (4.3%)	2 (3.9%)	0 (0%)	-
		Duration Days >60	N (%)	0 (0%)	0 (0%)	0 (0%)	0 (0%)	0 (0%)	-
General	Any	Symptom	N (%)	529 (92%)	138 (92%)	147 (96.7%)	181 (86.6%)	63 (94%)	0.006
		Persistence	N (%)	141 (27%)	5 (3.6%)	63 (42.9%)	56 (30.9%)	17 (27%)	<0.001
		Duration (in Days)	Median (IQR)	14 (7, 25.8)	10 (6.2, 15)	21 (13, 35)	14 (7, 26.2)	14 (7, 27.5)	<0.001
		Duration Days >30	N (%)	89 (17%)	7 (5.1%)	42 (28.6%)	29 (16%)	11 (17.5%)	-
		Duration Days >60	N (%)	21 (4%)	1 (0.7%)	14 (9.5%)	5 (2.8%)	1 (1.6%)	-
	Chills	Symptom	N (%)	296 (51%)	62 (41.3%)	95 (62.5%)	101 (48.3%)	38 (56.7%)	0.002
		Persistence	N (%)	11 (4%)	0 (0%)	4 (4.2%)	6 (5.9%)	1 (2.6%)	0.264
		Duration (in Days)	Median (IQR)	6 (3, 10)	4 (2, 5.8)	7 (3, 10)	7 (4, 14)	7 (3, 10)	<0.001
		Duration Days >30	N (%)	10 (3%)	0 (0%)	3 (3.2%)	5 (5%)	2 (5.3%)	-
		Duration Days >60	N (%)	3 (1%)	0 (0%)	1 (1.1%)	2 (2%)	0 (0%)	-
	Fever	Symptom	N (%)	344 (60%)	106 (70.7%)	113 (74.3%)	95 (45.5%)	30 (44.8%)	<0.001
		Persistence	N (%)	0 (0%)	0 (0%)	0 (0%)	0 (0%)	0 (0%)	-
		Duration (in Days)	Median (IQR)	6 (3, 10)	5 (3, 8)	7 (4, 14)	5 (3, 9.5)	7 (3, 14)	0.003
		Duration Days >30	N (%)	4 (1%)	0 (0%)	2 (1.8%)	2 (2.1%)	0 (0%)	-
		Duration Days >60	N (%)	0 (0%)	0 (0%)	0 (0%)	0 (0%)	0 (0%)	-
	Myalgia	Symptom	N (%)	333 (58%)	75 (50%)	105 (69.1%)	111 (53.1%)	42 (62.7%)	0.003
		Persistence	N (%)	49 (15%)	4 (5.3%)	12 (11.4%)	26 (23.4%)	7 (16.7%)	0.005
		Duration (in Days)	Median (IQR)	10 (5, 18.8)	7 (5, 15)	10 (4, 15)	12 (7, 25)	7 (5, 16.8)	0.014
		Duration Days >30	N (%)	32 (10%)	3 (4%)	9 (8.6%)	16 (14.4%)	4 (9.5%)	-
		Duration Days >60	N (%)	7 (2%)	1 (1.3%)	1 (1%)	4 (3.6%)	1 (2.4%)	-
	New or increased Fatigue	Symptom	N (%)	450 (78%)	95 (63.3%)	140 (92.1%)	160 (76.6%)	55 (82.1%)	<0.001
		Persistence	N (%)	119 (26%)	3 (3.2%)	55 (39.3%)	48 (30%)	13 (23.6%)	<0.001
		Duration (in Days)	Median (IQR)	14 (7, 28)	11 (7, 15.5)	21 (12, 35)	14 (7, 25.5)	14 (7, 28)	<0.001
		Duration Days >30	N (%)	83 (18%)	6 (6.3%)	40 (28.6%)	26 (16.2%)	11 (20%)	-
		Duration Days >60	N (%)	19 (4%)	1 (1.1%)	13 (9.3%)	4 (2.5%)	1 (1.8%)	-
	Unable to sleep lying down	Symptom	N (%)	164 (28%)	37 (24.7%)	47 (30.9%)	54 (25.8%)	26 (38.8%)	0.124
		Persistence	N (%)	16 (10%)	0 (0%)	7 (14.9%)	6 (11.1%)	3 (11.5%)	0.132
		Duration (in Days)	Median (IQR)	10 (5, 15)	7 (4, 14)	10 (5, 15.5)	8.5 (6, 14.8)	10 (7, 14)	0.424

		Duration Days >30	N (%)	8 (5%)	1 (2.7%)	4 (8.5%)	1 (1.9%)	2 (7.7%)	-
		Duration Days >60	N (%)	2 (1%)	0 (0%)	1 (2.1%)	1 (1.9%)	0 (0%)	-
Head, Eyes, Ears, Nose and Throat (HEENT)	Any	Symptom	N (%)	347 (60%)	97 (64.7%)	92 (60.5%)	109 (52.2%)	49 (73.1%)	0.009
		Persistence	N (%)	31 (9%)	4 (4.1%)	9 (9.8%)	15 (13.8%)	3 (6.1%)	0.092
		Duration (in Days)	Median (IQR)	7 (4, 14)	7 (4, 10)	10 (5, 15)	7 (4, 15)	7 (5, 14)	0.039
		Duration Days >30	N (%)	22 (6%)	2 (2.1%)	9 (9.8%)	9 (8.3%)	2 (4.1%)	-
		Duration Days >60	N (%)	6 (2%)	1 (1%)	2 (2.2%)	2 (1.8%)	1 (2%)	-
	Nasal congestion/Rhinorrhea	Symptom	N (%)	250 (43%)	58 (38.7%)	76 (50%)	76 (36.4%)	40 (59.7%)	0.001
		Persistence	N (%)	18 (7%)	1 (1.7%)	6 (7.9%)	9 (11.8%)	2 (5%)	0.145
		Duration (in Days)	Median (IQR)	7 (4, 14)	4 (3, 10)	10 (5, 14)	7 (4, 14)	7 (4.8, 10.2)	<0.001
		Duration Days >30	N (%)	14 (6%)	0 (0%)	8 (10.5%)	5 (6.6%)	1 (2.5%)	-
		Duration Days >60	N (%)	3 (1%)	0 (0%)	2 (2.6%)	1 (1.3%)	0 (0%)	-
	Sore throat	Symptom	N (%)	255 (44%)	80 (53.3%)	59 (38.8%)	79 (37.8%)	37 (55.2%)	0.003
		Persistence	N (%)	18 (7%)	3 (3.8%)	4 (6.8%)	9 (11.4%)	2 (5.4%)	0.289
		Duration (in Days)	Median (IQR)	7 (4, 13.5)	5 (3, 9.2)	5 (3, 14)	7 (4, 14)	7 (4, 10)	0.445
		Duration Days >30	N (%)	14 (5%)	2 (2.5%)	6 (10.2%)	5 (6.3%)	1 (2.7%)	-
		Duration Days >60	N (%)	4 (2%)	1 (1.2%)	1 (1.7%)	1 (1.3%)	1 (2.7%)	-
Respiratory	Any	Symptom	N (%)	511 (88%)	132 (88%)	135 (88.8%)	182 (87.1%)	62 (92.5%)	0.677
		Persistence	N (%)	151 (30%)	24 (18.2%)	49 (36.3%)	59 (32.4%)	19 (30.6%)	0.008
		Duration (in Days)	Median (IQR)	15 (7, 29)	15 (7, 23)	20.5 (10, 34.5)	12 (6, 22)	15 (10, 30)	<0.001
		Duration Days >30	N (%)	102 (20%)	21 (15.9%)	39 (28.9%)	27 (14.8%)	15 (24.2%)	-
		Duration Days >60	N (%)	27 (5%)	2 (1.5%)	14 (10.4%)	5 (2.7%)	6 (9.7%)	-
	Chest pain	Symptom	N (%)	267 (46%)	64 (42.7%)	59 (38.8%)	96 (45.9%)	48 (71.6%)	<0.001
		Persistence	N (%)	50 (19%)	7 (10.9%)	8 (13.6%)	20 (20.8%)	15 (31.2%)	0.032
		Duration (in Days)	Median (IQR)	10 (5, 20)	10 (5, 15)	10 (6, 15)	9 (5, 19)	13 (7, 30)	0.233
		Duration Days >30	N (%)	33 (12%)	6 (9.4%)	8 (13.6%)	8 (8.3%)	11 (22.9%)	-
		Duration Days >60	N (%)	9 (3%)	0 (0%)	3 (5.1%)	2 (2.1%)	4 (8.3%)	-
	Cough	Symptom	N (%)	385 (67%)	106 (70.7%)	111 (73%)	119 (56.9%)	49 (73.1%)	0.003
		Persistence	N (%)	74 (19%)	13 (12.3%)	30 (27%)	27 (22.7%)	4 (8.2%)	0.006
		Duration (in Days)	Median (IQR)	13 (6, 21)	11 (6, 20)	16 (7, 28.5)	10 (5, 19.5)	11 (6, 30)	0.031
		Duration Days >30	N (%)	58 (15%)	11 (10.4%)	23 (20.7%)	17 (14.3%)	7 (14.3%)	-
		Duration Days >60	N (%)	12 (3%)	2 (1.9%)	5 (4.5%)	4 (3.4%)	1 (2%)	-
	Dyspnea at rest	Symptom	N (%)	237 (41%)	63 (42%)	68 (44.7%)	75 (35.9%)	31 (46.3%)	0.265
		Persistence	N (%)	19 (8%)	1 (1.6%)	10 (14.7%)	7 (9.3%)	1 (3.2%)	0.032
		Duration (in Days)	Median (IQR)	8 (4, 15)	7 (3, 11.5)	11 (5, 19.2)	10 (5, 16)	7 (4.5, 16.5)	0.022
		Duration Days >30	N (%)	18 (8%)	1 (1.6%)	9 (13.2%)	6 (8%)	2 (6.5%)	-
		Duration Days >60	N (%)	4 (2%)	0 (0%)	2 (2.9%)	2 (2.7%)	0 (0%)	-
	Dyspnea with exertion	Symptom	N (%)	363 (63%)	102 (68%)	105 (69.1%)	119 (56.9%)	37 (55.2%)	0.029
		Persistence	N (%)	72 (20%)	9 (8.8%)	29 (27.6%)	28 (23.5%)	6 (16.2%)	0.004
		Duration (in Days)	Median (IQR)	11 (7, 21)	10 (6, 17)	15 (7, 30.2)	10 (6, 19.5)	10 (7, 18)	0.046
		Duration Days >30	N (%)	57 (16%)	11 (10.8%)	26 (24.8%)	15 (12.6%)	5 (13.5%)	-

		Duration Days >60	N (%)	16 (4%)	0 (0%)	11 (10.5%)	3 (2.5%)	2 (5.4%)	-
	Hemoptysis	Symptom	N (%)	24 (4%)	3 (2%)	10 (6.6%)	8 (3.8%)	3 (4.5%)	0.254
		Persistence	N (%)	1 (4%)	0 (0%)	0 (0%)	1 (12.5%)	0 (0%)	0.555
		Duration (in Days)	Median (IQR)	4 (2, 7)	14 (7.5, 17)	2.5 (2, 6.2)	4.5 (2.8, 7)	5 (4, 5.5)	0.714
		Duration Days >30	N (%)	0 (0%)	0 (0%)	0 (0%)	0 (0%)	0 (0%)	-
		Duration Days >60	N (%)	0 (0%)	0 (0%)	0 (0%)	0 (0%)	0 (0%)	-
	Increased sputum production	Symptom	N (%)	161 (28%)	39 (26%)	48 (31.6%)	48 (23%)	26 (38.8%)	0.051
		Persistence	N (%)	17 (11%)	2 (5.1%)	3 (6.2%)	10 (20.8%)	2 (7.7%)	0.051
		Duration (in Days)	Median (IQR)	8 (4, 14)	7 (3.5, 13)	7 (4, 14)	9.5 (4.8, 17.5)	10.5 (6.2, 14)	0.248
		Duration Days >30	N (%)	13 (8%)	1 (2.6%)	4 (8.3%)	6 (12.5%)	2 (7.7%)	-
		Duration Days >60	N (%)	3 (2%)	0 (0%)	0 (0%)	2 (4.2%)	1 (3.8%)	-
Skin	New Skin findings	Symptom	N (%)	39 (7%)	2 (1.3%)	20 (13.2%)	10 (4.8%)	7 (10.4%)	<0.001
		Persistence	N (%)	12 (31%)	0 (0%)	8 (40%)	2 (20%)	2 (28.6%)	0.522
		Duration (in Days)	Median (IQR)	10.5 (6.2, 21)	9 (8.5, 9.5)	10.5 (5.8, 23.2)	14 (5, 24)	14 (7, 17.5)	0.991
		Duration Days >30	N (%)	4 (10%)	0 (0%)	3 (15%)	1 (10%)	0 (0%)	-
		Duration Days >60	N (%)	1 (3%)	0 (0%)	0 (0%)	1 (10%)	0 (0%)	-

Supplemental Table 9. Summary of persistence beyond acute COVID-19 resolution and symptom duration by COVID-19 severity.

Body System	Symptom	Outcome	Statistics	Total	Symptomatic, no O2 requirement [NOR]	Symptomatic, non-invasive O2 requirement [NIOR]	Symptomatic, invasive O2 requirement [IOR]	p-value
Any System	Any Symptom	Symptom	N (%)	578 (100%)	360 (100%)	174 (100%)	44 (100%)	-
		Persistence	N (%)	251 (43%)	144 (40%)	88 (50.6%)	19 (43.2%)	0.069
		Duration (in Days)	Median (IQR)	20 (11, 35)	16 (8, 30)	26 (15, 41)	24.5 (14.8, 36)	<0.001
		Duration Days >30	N (%)	163 (28%)	84 (23.3%)	63 (36.2%)	16 (36.4%)	-
		Duration Days >60	N (%)	46 (8%)	22 (6.1%)	21 (12.1%)	3 (6.8%)	-
Central Nervous System (CNS)	Any	Symptom	N (%)	496 (86%)	319 (88.6%)	146 (83.9%)	31 (70.5%)	0.003
		Persistence	N (%)	114 (23%)	67 (21%)	43 (29.5%)	4 (12.9%)	0.051
		Duration (in Days)	Median (IQR)	11 (7, 21)	10 (6, 19.8)	14 (7.2, 25.8)	13 (9, 21)	0.004
		Duration Days >30	N (%)	71 (14%)	39 (12.2%)	26 (17.8%)	6 (19.4%)	-
		Duration Days >60	N (%)	23 (5%)	12 (3.8%)	9 (6.2%)	2 (6.5%)	-
	Altered mental status/confusion	Symptom	N (%)	94 (16%)	44 (12.2%)	35 (20.1%)	15 (34.1%)	<0.001
		Persistence	N (%)	18 (19%)	5 (11.4%)	12 (34.3%)	1 (6.7%)	0.015
		Duration (in Days)	Median (IQR)	7 (3, 14)	5.5 (2, 13.2)	12 (3, 27)	7 (3, 12.5)	0.187
		Duration Days >30	N (%)	12 (13%)	4 (9.1%)	6 (17.1%)	2 (13.3%)	-
		Duration Days >60	N (%)	7 (7%)	3 (6.8%)	3 (8.6%)	1 (6.7%)	-
	Anosmia/Hyposmia	Symptom	N (%)	314 (54%)	208 (57.8%)	88 (50.6%)	18 (40.9%)	0.052
		Persistence	N (%)	49 (16%)	31 (14.9%)	16 (18.2%)	2 (11.1%)	0.671
		Duration (in Days)	Median (IQR)	10 (6, 18)	10 (6, 17)	12 (7, 20.5)	10 (3.2, 33.2)	0.281
		Duration Days >30	N (%)	33 (11%)	18 (8.7%)	10 (11.4%)	5 (27.8%)	-
		Duration Days >60	N (%)	7 (2%)	4 (1.9%)	2 (2.3%)	1 (5.6%)	-
	Headache	Symptom	N (%)	374 (65%)	255 (70.8%)	100 (57.5%)	19 (43.2%)	<0.001
		Persistence	N (%)	63 (17%)	38 (14.9%)	23 (23%)	2 (10.5%)	0.140
		Duration (in Days)	Median (IQR)	8 (4, 14)	7 (4, 14)	10 (5, 20.2)	12 (4.5, 16)	0.015
		Duration Days >30	N (%)	36 (10%)	19 (7.5%)	16 (16%)	1 (5.3%)	-
		Duration Days >60	N (%)	12 (3%)	7 (2.7%)	5 (5%)	0 (0%)	-
	Hypogeusia	Symptom	N (%)	351 (61%)	227 (63.1%)	105 (60.3%)	19 (43.2%)	0.039
		Persistence	N (%)	47 (13%)	25 (11%)	20 (19%)	2 (10.5%)	0.126
		Duration (in Days)	Median (IQR)	10 (6, 17.2)	8.5 (5.2, 15)	13 (7, 20)	10 (4, 23)	0.128
		Duration Days >30	N (%)	27 (8%)	16 (7%)	7 (6.7%)	4 (21.1%)	-
		Duration Days >60	N (%)	4 (1%)	3 (1.3%)	0 (0%)	1 (5.3%)	-
Gastrointestinal (GIT)	Any	Symptom	N (%)	397 (69%)	244 (67.8%)	128 (73.6%)	25 (56.8%)	0.084
		Persistence	N (%)	46 (12%)	25 (10.2%)	17 (13.3%)	4 (16%)	0.532
		Duration (in Days)	Median (IQR)	7.5 (4, 14)	7 (4, 14)	10 (5, 14)	10 (6, 20)	0.070
		Duration Days >30	N (%)	23 (6%)	13 (5.3%)	10 (7.8%)	0 (0%)	-
		Duration Days >60	N (%)	6 (2%)	3 (1.2%)	3 (2.3%)	0 (0%)	-
	Abdominal pain	Symptom	N (%)	99 (17%)	67 (18.6%)	26 (14.9%)	6 (13.6%)	0.467

		Persistence	N (%)	10 (10%)	5 (7.5%)	4 (15.4%)	1 (16.7%)	0.450
		Duration (in Days)	Median (IQR)	5 (3, 10.5)	5 (3, 11.5)	5 (3, 9.8)	5 (5, 8.8)	0.911
		Duration Days >30	N (%)	3 (3%)	2 (3%)	1 (3.8%)	0 (0%)	-
		Duration Days >60	N (%)	2 (2%)	2 (3%)	0 (0%)	0 (0%)	-
	Anorexia	Symptom	N (%)	270 (47%)	160 (44.4%)	89 (51.1%)	21 (47.7%)	0.343
		Persistence	N (%)	16 (6%)	10 (6.2%)	5 (5.6%)	1 (4.8%)	0.953
		Duration (in Days)	Median (IQR)	10 (6, 14)	8 (5, 14)	10 (7, 14)	10 (4, 13)	0.393
		Duration Days >30	N (%)	16 (6%)	9 (5.6%)	7 (7.9%)	0 (0%)	-
		Duration Days >60	N (%)	2 (1%)	0 (0%)	2 (2.2%)	0 (0%)	-
	Diarrhea	Symptom	N (%)	229 (40%)	137 (38.1%)	82 (47.1%)	10 (22.7%)	0.008
		Persistence	N (%)	20 (9%)	9 (6.6%)	10 (12.2%)	1 (10%)	0.357
		Duration (in Days)	Median (IQR)	5 (2, 8)	4 (2, 7)	5.5 (2.2, 12.8)	5 (3.5, 6.8)	0.254
		Duration Days >30	N (%)	6 (3%)	2 (1.5%)	4 (4.9%)	0 (0%)	-
		Duration Days >60	N (%)	3 (1%)	2 (1.5%)	1 (1.2%)	0 (0%)	-
	Nausea/Vomiting	Symptom	N (%)	161 (28%)	97 (26.9%)	53 (30.5%)	11 (25%)	0.633
		Persistence	N (%)	10 (6%)	5 (5.2%)	3 (5.7%)	2 (18.2%)	0.232
		Duration (in Days)	Median (IQR)	5 (2, 9)	4 (2, 7)	6 (2, 10)	4 (2.5, 9.5)	0.489
		Duration Days >30	N (%)	4 (2%)	2 (2.1%)	2 (3.8%)	0 (0%)	-
		Duration Days >60	N (%)	0 (0%)	0 (0%)	0 (0%)	0 (0%)	-
General	Any	Symptom	N (%)	529 (92%)	322 (89.4%)	163 (93.7%)	44 (100%)	0.028
		Persistence	N (%)	141 (27%)	76 (23.6%)	51 (31.3%)	14 (31.8%)	0.141
		Duration (in Days)	Median (IQR)	14 (7, 25.8)	13 (7, 21)	15 (10, 30)	19 (9.5, 31)	<0.001
		Duration Days >30	N (%)	89 (17%)	43 (13.4%)	34 (20.9%)	12 (27.3%)	-
		Duration Days >60	N (%)	21 (4%)	8 (2.5%)	11 (6.7%)	2 (4.5%)	-
	Chills	Symptom	N (%)	296 (51%)	183 (50.8%)	94 (54%)	19 (43.2%)	0.426
		Persistence	N (%)	11 (4%)	6 (3.3%)	4 (4.3%)	1 (5.3%)	0.860
		Duration (in Days)	Median (IQR)	6 (3, 10)	5 (3, 9)	6.5 (3, 11.8)	10 (5, 11)	0.101
		Duration Days >30	N (%)	10 (3%)	4 (2.2%)	6 (6.4%)	0 (0%)	-
		Duration Days >60	N (%)	3 (1%)	2 (1.1%)	1 (1.1%)	0 (0%)	-
	Fever	Symptom	N (%)	344 (60%)	194 (53.9%)	113 (64.9%)	37 (84.1%)	<0.001
		Persistence	N (%)	0 (0%)	0 (0%)	0 (0%)	0 (0%)	-
		Duration (in Days)	Median (IQR)	6 (3, 10)	5 (3, 10)	7 (4, 12)	7 (4, 10)	0.006
		Duration Days >30	N (%)	4 (1%)	2 (1%)	2 (1.8%)	0 (0%)	-
		Duration Days >60	N (%)	0 (0%)	0 (0%)	0 (0%)	0 (0%)	-
	Myalgia	Symptom	N (%)	333 (58%)	212 (58.9%)	96 (55.2%)	25 (56.8%)	0.713
		Persistence	N (%)	49 (15%)	23 (10.8%)	18 (18.8%)	8 (32%)	0.008
		Duration (in Days)	Median (IQR)	10 (5, 18.8)	7 (4, 14.8)	14 (7, 25)	15.5 (7, 27)	<0.001
		Duration Days >30	N (%)	32 (10%)	16 (7.5%)	11 (11.5%)	5 (20%)	-
		Duration Days >60	N (%)	7 (2%)	1 (0.5%)	5 (5.2%)	1 (4%)	-
	New or increased Fatigue	Symptom	N (%)	450 (78%)	272 (75.6%)	141 (81%)	37 (84.1%)	0.210
		Persistence	N (%)	119 (26%)	64 (23.5%)	43 (30.5%)	12 (32.4%)	0.216
		Duration (in Days)	Median (IQR)	14 (7, 28)	14 (7, 21)	19 (10, 30)	23.5 (10, 31.5)	<0.001
		Duration Days >30	N (%)	83 (18%)	38 (14%)	33 (23.4%)	12 (32.4%)	-
		Duration Days >60	N (%)	19 (4%)	7 (2.6%)	10 (7.1%)	2 (5.4%)	-
	Unable to sleep lying down	Symptom	N (%)	164 (28%)	83 (23.1%)	65 (37.4%)	16 (36.4%)	0.001

		Persistence	N (%)	16 (10%)	9 (10.8%)	7 (10.8%)	0 (0%)	0.383
		Duration (in Days)	Median (IQR)	10 (5, 15)	7 (4, 14)	10 (7, 15)	10 (6.5, 15)	0.014
		Duration Days >30	N (%)	8 (5%)	3 (3.6%)	5 (7.7%)	0 (0%)	-
		Duration Days >60	N (%)	2 (1%)	2 (2.4%)	0 (0%)	0 (0%)	-
Head, Eyes, Ears, Nose and Throat (HEENT)	Any	Symptom	N (%)	347 (60%)	232 (64.4%)	95 (54.6%)	20 (45.5%)	0.011
		Persistence	N (%)	31 (9%)	19 (8.2%)	10 (10.5%)	2 (10%)	0.786
		Duration (in Days)	Median (IQR)	7 (4, 14)	7 (4, 14)	10 (5, 19.5)	7 (4.8, 10.5)	0.073
		Duration Days >30	N (%)	22 (6%)	10 (4.3%)	11 (11.6%)	1 (5%)	-
		Duration Days >60	N (%)	6 (2%)	2 (0.9%)	4 (4.2%)	0 (0%)	-
	Nasal congestion/Rhinorrhea	Symptom	N (%)	250 (43%)	169 (46.9%)	67 (38.5%)	14 (31.8%)	0.051
		Persistence	N (%)	18 (7%)	12 (7.1%)	5 (7.5%)	1 (7.1%)	0.995
		Duration (in Days)	Median (IQR)	7 (4, 14)	7 (4, 14)	7 (3, 14.5)	6 (4.2, 10)	0.918
		Duration Days >30	N (%)	14 (6%)	7 (4.1%)	6 (9%)	1 (7.1%)	-
		Duration Days >60	N (%)	3 (1%)	2 (1.2%)	1 (1.5%)	0 (0%)	-
	Sore throat	Symptom	N (%)	255 (44%)	171 (47.5%)	68 (39.1%)	16 (36.4%)	0.104
		Persistence	N (%)	18 (7%)	11 (6.4%)	6 (8.8%)	1 (6.2%)	0.802
		Duration (in Days)	Median (IQR)	7 (4, 13.5)	5 (3, 10)	7.5 (5, 18.2)	7 (4.8, 8.5)	0.002
		Duration Days >30	N (%)	14 (5%)	5 (2.9%)	9 (13.2%)	0 (0%)	-
		Duration Days >60	N (%)	4 (2%)	1 (0.6%)	3 (4.4%)	0 (0%)	-
Respiratory	Any	Symptom	N (%)	511 (88%)	300 (83.3%)	169 (97.1%)	42 (95.5%)	<0.001
		Persistence	N (%)	151 (30%)	77 (25.7%)	62 (36.7%)	12 (28.6%)	0.042
		Duration (in Days)	Median (IQR)	15 (7, 29)	11 (6, 21)	20 (11, 35)	21 (11.2, 30)	<0.001
		Duration Days >30	N (%)	102 (20%)	44 (14.7%)	48 (28.4%)	10 (23.8%)	-
		Duration Days >60	N (%)	27 (5%)	14 (4.7%)	12 (7.1%)	1 (2.4%)	-
	Chest pain	Symptom	N (%)	267 (46%)	158 (43.9%)	91 (52.3%)	18 (40.9%)	0.144
		Persistence	N (%)	50 (19%)	30 (19%)	18 (19.8%)	2 (11.1%)	0.684
		Duration (in Days)	Median (IQR)	10 (5, 20)	7 (5, 15)	12 (7, 25)	12 (5.5, 19.2)	0.028
		Duration Days >30	N (%)	33 (12%)	16 (10.1%)	15 (16.5%)	2 (11.1%)	-
		Duration Days >60	N (%)	9 (3%)	7 (4.4%)	2 (2.2%)	0 (0%)	-
	Cough	Symptom	N (%)	385 (67%)	223 (61.9%)	129 (74.1%)	33 (75%)	0.009
		Persistence	N (%)	74 (19%)	39 (17.5%)	29 (22.5%)	6 (18.2%)	0.513
		Duration (in Days)	Median (IQR)	13 (6, 21)	10 (5, 20)	15 (9, 29)	17 (8, 26)	<0.001
		Duration Days >30	N (%)	58 (15%)	25 (11.2%)	27 (20.9%)	6 (18.2%)	-
		Duration Days >60	N (%)	12 (3%)	5 (2.2%)	6 (4.7%)	1 (3%)	-
	Dyspnea at rest	Symptom	N (%)	237 (41%)	99 (27.5%)	107 (61.5%)	31 (70.5%)	<0.001
		Persistence	N (%)	19 (8%)	8 (8.1%)	9 (8.4%)	2 (6.5%)	0.939
		Duration (in Days)	Median (IQR)	8 (4, 15)	6 (4, 14)	10 (5, 17.5)	11 (7, 20.5)	0.004
		Duration Days >30	N (%)	18 (8%)	10 (10.1%)	7 (6.5%)	1 (3.2%)	-
		Duration Days >60	N (%)	4 (2%)	3 (3%)	0 (0%)	1 (3.2%)	-
	Dyspnea with exertion	Symptom	N (%)	363 (63%)	168 (46.7%)	156 (89.7%)	39 (88.6%)	<0.001
		Persistence	N (%)	72 (20%)	30 (17.9%)	34 (21.8%)	8 (20.5%)	0.670
		Duration (in Days)	Median (IQR)	11 (7, 21)	10 (5, 15.5)	15 (7, 28)	11 (7, 25)	0.002
		Duration Days >30	N (%)	57 (16%)	22 (13.1%)	30 (19.2%)	5 (12.8%)	-
		Duration Days >60	N (%)	16 (4%)	9 (5.4%)	6 (3.8%)	1 (2.6%)	-

	Hemoptysis	Symptom	N (%)	24 (4%)	8 (2.2%)	12 (6.9%)	4 (9.1%)	0.009
		Persistence	N (%)	1 (4%)	1 (12.5%)	0 (0%)	0 (0%)	0.352
		Duration (in Days)	Median (IQR)	4 (2, 7)	3 (1.8, 4)	6 (2.8, 10.2)	4 (2, 6.2)	0.235
		Duration Days >30	N (%)	0 (0%)	0 (0%)	0 (0%)	0 (0%)	-
		Duration Days >60	N (%)	0 (0%)	0 (0%)	0 (0%)	0 (0%)	-
	Increased sputum production	Symptom	N (%)	161 (28%)	90 (25%)	58 (33.3%)	13 (29.5%)	0.127
		Persistence	N (%)	17 (11%)	7 (7.8%)	10 (17.2%)	0 (0%)	0.082
		Duration (in Days)	Median (IQR)	8 (4, 14)	7 (4, 11)	12 (7, 20.8)	6 (3, 14)	<0.001
		Duration Days >30	N (%)	13 (8%)	6 (6.7%)	6 (10.3%)	1 (7.7%)	-
		Duration Days >60	N (%)	3 (2%)	1 (1.1%)	1 (1.7%)	1 (7.7%)	-
Skin	New Skin findings	Symptom	N (%)	39 (7%)	25 (6.9%)	11 (6.3%)	3 (6.8%)	0.964
		Persistence	N (%)	12 (31%)	6 (24%)	5 (45.5%)	1 (33.3%)	0.436
		Duration (in Days)	Median (IQR)	10.5 (6.2, 21)	9 (5.8, 21)	10 (6, 21)	21 (16.5, 40.5)	0.327
		Duration Days >30	N (%)	4 (10%)	3 (12%)	0 (0%)	1 (33.3%)	-
		Duration Days >60	N (%)	1 (3%)	1 (4%)	0 (0%)	0 (0%)	-

Figures

Supplemental Figure 1. Median Symptom Duration, By Region and Symptom Severity.

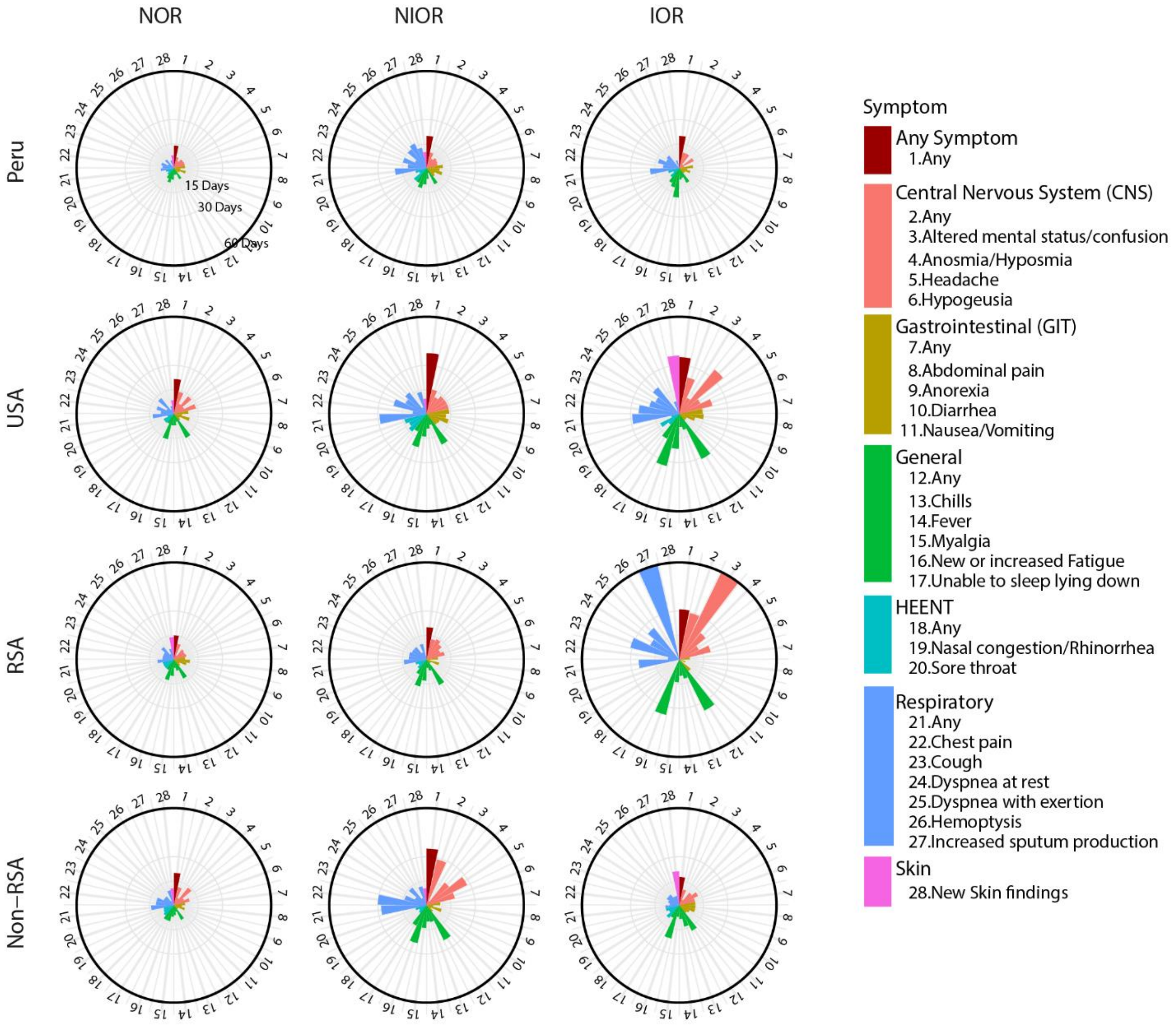

Supplemental Figure 2. Symptom Persistence Beyond Acute COVID-19 Resolution and Symptom Duration by COVID-19 Severity.

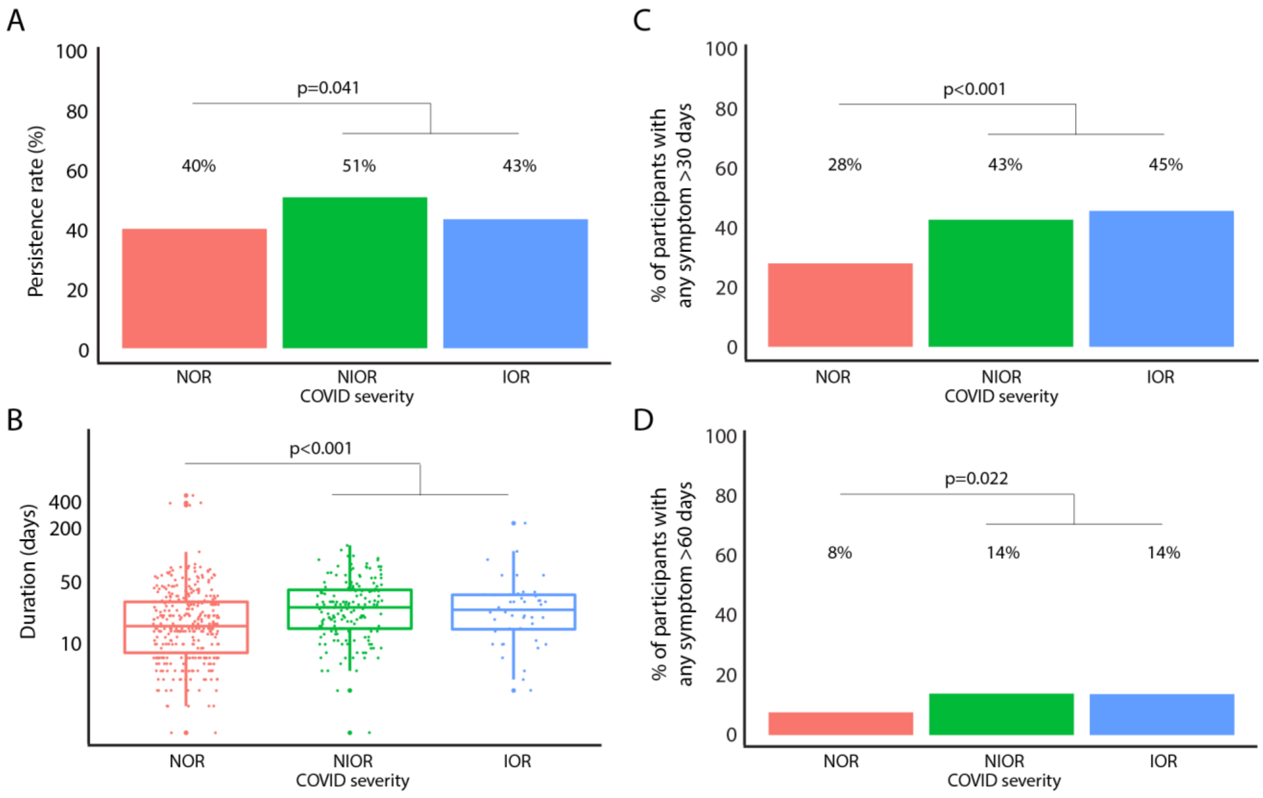

Supplement: Online Supplementary Document [file jogh-13-06020-s001.pdf]
